# Structure-Prior-Guided Multi-Stage Cross-Modal Collaborative Network for RGB-D Semantic Segmentation

**DOI:** 10.3390/jimaging12080394

**Published:** 2026-08-20

**Authors:** Yifan Yu, Zhiwei Zhong, Fan Min, Song Deng

**Affiliations:** School of Computer Science and Software Engineering, Southwest Petroleum University, Chengdu 610500, China; yuyifan@swpu.edu.cn (Y.Y.); 202421000539@stu.swpu.edu.cn (Z.Z.); 202421000516@stu.swpu.edu.cn (S.D.)

**Keywords:** red–green–blue and depth (RGB-D) semantic segmentation, structure prior, cross-modal correction, depth reliability, multi-scale fusion

## Abstract

Red–green–blue and depth (RGB-D) semantic segmentation combines appearance cues from RGB images with geometric information from depth maps, but sensor noise, missing measurements, and boundary-inconsistent depth responses can introduce conflicting evidence during cross-modal fusion. We propose the Structure-Prior-Guided Network (SPGNet), a dual-branch, multi-stage framework that follows a correction-before-fusion strategy. At each feature scale, SPGNet estimates a learned structure prior from cross-modal agreement and discrepancy. The Cross-Modal Correction Module (CCM) uses this prior to regulate bidirectional information transfer, suppressing unreliable responses while retaining complementary cues. The Dual-branch Enhancement Fusion Module (DEF) then enhances the corrected RGB and depth features and integrates them through shared-representation-guided interaction, after which a lightweight multi-scale decoder produces the segmentation output. Under a unified training and evaluation protocol, SPGNet achieved three-run mean Intersection over Union (mIoU) scores of 50.845% on NYU Depth V2 and 48.457% on SUN RGB-D. Compared with the best reproduced baseline on each dataset, SPGNet improved mean mIoU by 2.111 and 0.899 percentage points, respectively. These results suggest that separating reliability-oriented correction from multimodal fusion can limit the propagation of unreliable cross-modal responses and improve indoor RGB-D semantic segmentation performance.

## 1. Introduction

Semantic segmentation, which assigns a semantic label to each image pixel, is a fundamental problem in indoor scene understanding and supports applications such as robotic navigation, scene reconstruction, and human–environment interaction [1,2,3]. Red–green–blue (RGB) images provide rich color, texture, and semantic context, but RGB-only parsing is often degraded by weak texture, illumination variation, occlusion, and cluttered indoor layouts. With the increasing availability of depth sensors, RGB and depth (RGB-D) semantic segmentation has become an effective way to improve indoor parsing by introducing geometric information complementary to RGB appearance [4,5,6]. Depth maps describe object contours, spatial layout, and structural discontinuities, which are especially useful for distinguishing regions that are visually similar but geometrically different [7,8].

**Figure 1:**
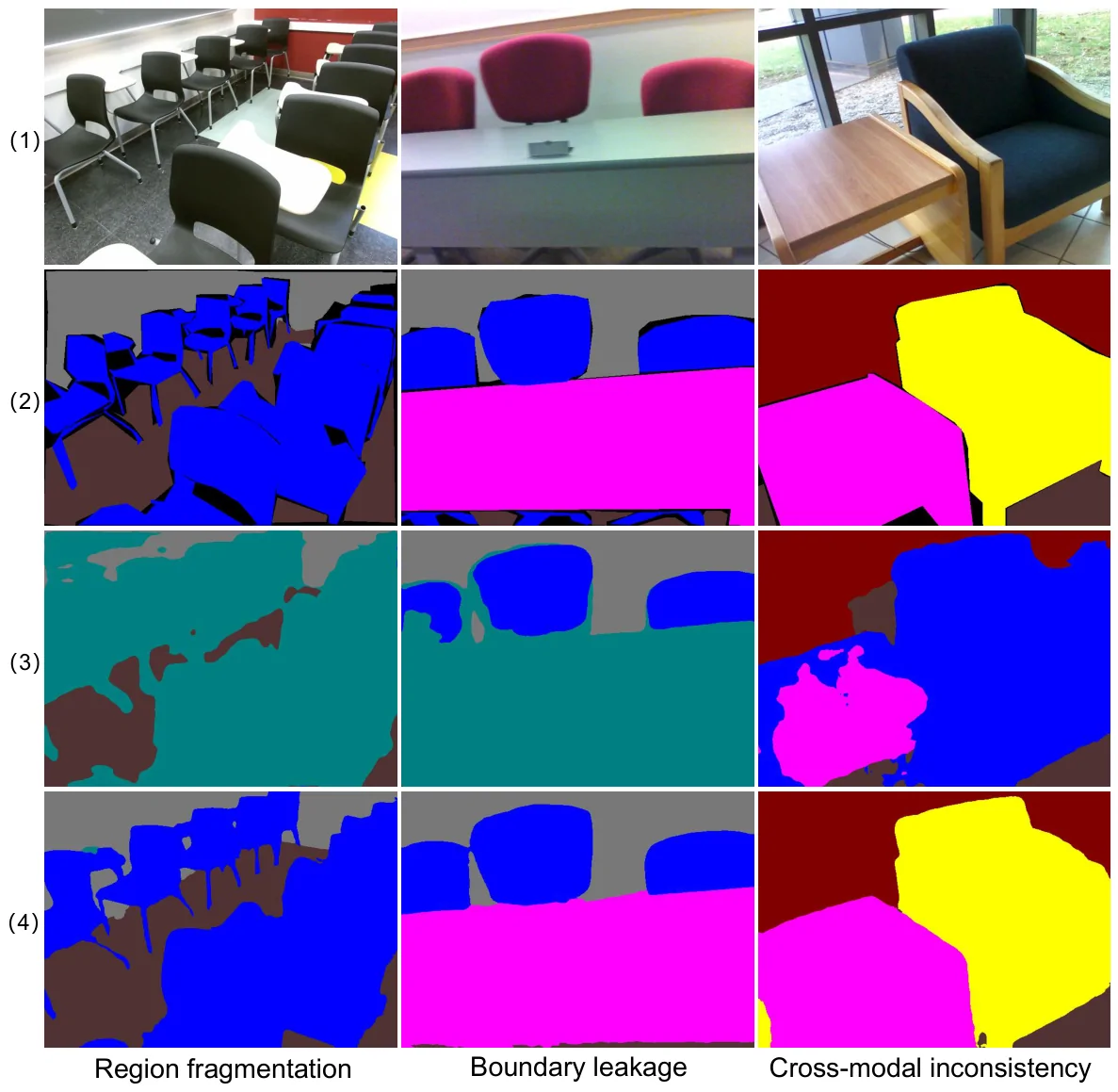
Qualitative motivation examples from NYU Depth V2. The three columns illustrate region fragmentation, boundary leakage, and cross-modal inconsistency, respectively. Rows (1)–(4) show the red–green–blue (RGB) images, ground-truth annotations, Direct Fusion predictions, and Structure-Prior-Guided Network (SPGNet) predictions, respectively.

However, introducing depth information does not automatically lead to better segmentation. The main difficulty lies in both modality heterogeneity and modality reliability. RGB and depth describe the same scene from different physical perspectives: RGB features emphasize photometric appearance, whereas depth features encode geometric structure and relative spatial layout. At the same time, depth maps captured in indoor scenes may contain sensor noise, missing values, local misalignment, and boundary-inconsistent responses [4,9,10]. If these unreliable responses are directly exchanged or fused with RGB features, local conflicts between appearance and geometry can be propagated into the final representation, causing fragmented object regions, blurred boundaries, and semantic leakage across adjacent categories. Figure 1 illustrates this issue through three representative failure modes of Direct Fusion: region fragmentation, boundary leakage, and cross-modal inconsistency. Here, Direct Fusion denotes a simple RGB-D feature integration baseline without the proposed pre-fusion correction. In the region-fragmentation example (left column), Direct Fusion fails to preserve the individual chair regions and the gaps between adjacent objects, whereas SPGNet recovers a more coherent arrangement. In the boundary-leakage example (middle column), Direct Fusion confuses much of the table region and introduces errors near the chair–table boundary; SPGNet produces a cleaner separation between these categories. In the cross-modal-inconsistency example (right column), Direct Fusion yields substantial class confusion between the table and chair regions, whereas SPGNet restores their dominant semantic regions more consistently with the annotation. Although some annotation boundaries are relatively coarse, these region-level observations are consistent with the interpretation that pre-fusion correction can limit the propagation of conflicting cross-modal responses; the controlled ablation in Section 4.4 provides the corresponding quantitative evidence.

Existing RGB-D semantic segmentation methods have explored this problem from several directions. Early and convolutional neural network (CNN)-based methods commonly adopt dual-branch encoders, residual fusion, gated propagation, context aggregation, or geometry-aware convolution to combine RGB appearance and depth geometry [5,6,9,11]. Recent Transformer-based and hybrid methods further introduce token interaction, cross-modal feature rectification, asymmetric modality modeling, RGB-D representation pretraining, primary-modality guidance, and depth-guided self-attention [10,12,13,14,15,16]. These studies show that RGB and depth should not be treated as two identical inputs, and that their complementary information must be selected and aligned carefully. Although these methods have significantly improved RGB-D feature interaction, reliability modeling is usually coupled with the fusion or interaction process itself. In such designs, unreliable source-modality responses may be injected into the target branch before their local compatibility is explicitly estimated. This motivates a pre-fusion mechanism that first models feature-level RGB–depth agreement and discrepancy, and then uses the resulting structure prior to regulate the direction and strength of cross-modal transfer.

Motivated by this, this paper proposes the Structure-Prior-Guided Network (SPGNet), a dual-branch multi-stage framework for RGB-D semantic segmentation. The key idea of SPGNet is to decouple reliability-oriented cross-modal correction from representation-oriented multimodal fusion. At each feature scale, SPGNet first estimates a learned feature-level structure prior from RGB–depth consistency and discrepancy cues. Instead of relying on externally supervised edge maps or ground-truth structural labels, this prior is learned in the encoded feature space and reflects local cross-modal compatibility. Guided by this prior, the Cross-Modal Correction Module (CCM) performs gated bidirectional correction to regulate cross-modal transfer before fusion. The Dual-branch Enhancement Fusion Module (DEF) then enhances the corrected modality-specific features and integrates them through shared-representation-guided interaction. Finally, a lightweight multi-scale decoder aggregates the fused features for dense prediction.

This separation enables the network to reduce the influence of locally inconsistent modality responses before feature integration, while preserving both appearance semantics and geometric structure for indoor scene parsing. Experiments on NYU Depth V2 and SUN RGB-D show that SPGNet consistently improves over representative reproduced RGB-D segmentation baselines under the unified evaluation protocol, and ablation studies further support the complementary roles of CCM and DEF.

The main contributions of this paper are summarized as follows:

- We propose SPGNet, a multi-stage RGB-D semantic segmentation framework that decouples reliability-oriented cross-modal correction from representation-oriented multimodal fusion.
- We design CCM to estimate a learned feature-level structure prior from RGB–depth consistency and discrepancy cues, and to use this prior for gated bidirectional cross-modal correction before fusion.
- We design DEF to enhance the corrected RGB and depth features and to integrate them through shared-representation-guided interaction, preserving modality-specific cues while forming a unified multimodal representation.

## 2. Related Work

### 2.1. CNN-Based RGB-D Fusion and Geometric Modeling Methods

Early related studies explored perceptual organization, multi-task geometry prediction, and explicit RGB-D constraints before deep bimodal fusion became dominant [17,18,19]. Later, RGB-D semantic segmentation methods commonly used convolutional or recurrent dual-branch architectures to combine RGB appearance with depth geometry. LSTM-CF [20] unifies contextual modeling and RGB-D fusion with LSTMs, while FuseNet [5] injects depth features into an RGB encoder–decoder network, showing that geometric information can improve indoor scene parsing. Common and specific feature learning, STD2P-based pooling, graph-based 3D relation modeling, multi-level residual fusion, gated fusion, and residual encoder–decoder designs further enriched RGB-D feature extraction and interaction [6,21,22,23,24,25]. Depth-aware CNN [7] introduces depth-aware operators, and PAD-Net [26] uses auxiliary prediction and distillation to strengthen dense representation learning. These methods demonstrate that depth can complement RGB features, while leaving room for complementary designs that explicitly estimate local source-feature conflicts before cross-modal transfer.

Other CNN-based studies place more emphasis on geometric structure, context, attention, and spatial guidance. GAD [8] distills geometry-aware embeddings and uses geometric propagation for indoor segmentation. ACNet [27] and CANet [28] model RGB-D complementarity through attention-based or collaborative feature interaction. Malleable 2.5D convolution [29] learns receptive fields along the depth axis for RGB-D scene parsing, while SCN [30] and Zig-Zag Network [31] study switchable context and cross-modal interaction for RGB-D segmentation. CEN [32] explores adaptive multimodal fusion through channel exchanging, and SA-Gate [9] performs bidirectional cross-modality propagation with separation-and-aggregation gating.

More recent CNN-based methods further investigate gated collaboration, depth enhancement, efficient encoding, and local structural operators. SGNet [33] and ESANet [34] study spatial guidance and efficient RGB-D encoding, while ShapeConv [11] introduces shape-aware convolution for local geometric structure. MGCNet [35] studies gated collaboration with multilevel feature interaction. PGDENet [36] introduces progressive guided fusion and depth enhancement for indoor scene parsing, SGACNet [37] explores spatial-information-guided adaptive context modeling, PDCNet [38] uses pixel-difference convolution, and DCANet [39] introduces differential convolution attention. These methods strengthen the use of geometric cues and cross-modal interaction from different perspectives. The present work is motivated by a complementary question: when depth features contain unreliable responses or local conflicts with RGB appearance, feature-level structural agreement and discrepancy can be used to model local source-feature reliability before cross-modal transfer. This motivates the pre-fusion correction design used in this work.

### 2.2. Transformer-Based RGB-D Semantic Segmentation Methods

A number of recent RGB-D segmentation methods adopt Transformer or hybrid architectures to model long-range dependencies and cross-modal correspondences. TransD-Fusion [40] studies Transformer fusion for indoor RGB-D semantic segmentation. TokenFusion [12] identifies uninformative tokens and replaces them with projected cross-modal features. UCTNet [41] introduces uncertainty-aware cross-modal Transformer modeling for indoor RGB-D segmentation. CMX [10] uses a Cross-Modal Feature Rectification Module and a Feature Fusion Module to align and fuse RGB-X features, including RGB-D and other modality combinations. DFormer [14] introduces RGB-D pre-training and RGB-D-specific blocks to improve depth-aware representation learning, and PrimKD [15] uses primary-modality-guided multimodal fusion to improve RGB-D representation learning.

Other recent methods further refine arbitrary-modal robustness, asymmetric design, efficient adaptation, and geometry-aware attention. Beyond RGB-D, arbitrary-modal segmentation [42] studies robust semantic parsing when different auxiliary modalities are available. AsymFormer [13] adopts asymmetric backbones and attention-guided feature selection to balance accuracy and efficiency, while DPLNet [43] keeps the RGB-pretrained backbone fixed and performs multimodal adaptation through prompt and adapter modules. CDMANet [44] models same-scale cross-modal correlations with central-difference mutual attention and uses a composite decoder for cross-scale fusion. DFormerv2 [16] treats depth as a geometric prior for self-attention and explicitly models depth-guided attention weights, and parameter-efficient symmetrical RGB-X modeling [45] further studies scalable multimodal semantic parsing. Together, these studies illustrate different uses of global interaction, modality-aware adaptation, and geometry-related attention mechanisms in RGB-D and broader RGB-X segmentation.

The proposed method is complementary to these studies. Many Transformer-based methods focus on global representation learning, token-level interaction, or fusion-stage module design. Some of them include rectification or token selection; in this subsection, pre-fusion local reliability filtering refers specifically to estimating feature-level agreement and discrepancy before cross-modal transfer. SPGNet focuses on this pre-fusion stage: CCM estimates a structure-prior map from relative edge agreement and feature discrepancy and uses the structure-prior map to filter cross-modal transfer; DEF then enhances and fuses the corrected modality features across stages.

## 3. Materials and Methods

### 3.1. Overall Framework

Given an RGB image $I_{r}\in R^{3\times H\times W}$ and a depth map $I_{d}\in R^{1\times H\times W}$, the depth channel is replicated to obtain ${\tilde{I}}_{d}\in R^{3\times H\times W}$. SPGNet predicts

(1) $$Y=\Phi (I_{r},{\tilde{I}}_{d}),$$

where $Y\in R^{K\times H\times W}$ contains the final logits and *K* includes the dataset-specific ignored label index. Supervision uses $Y^{gt}$, with unlabeled pixels excluded as defined in Section 3.5.

**Figure 2:**
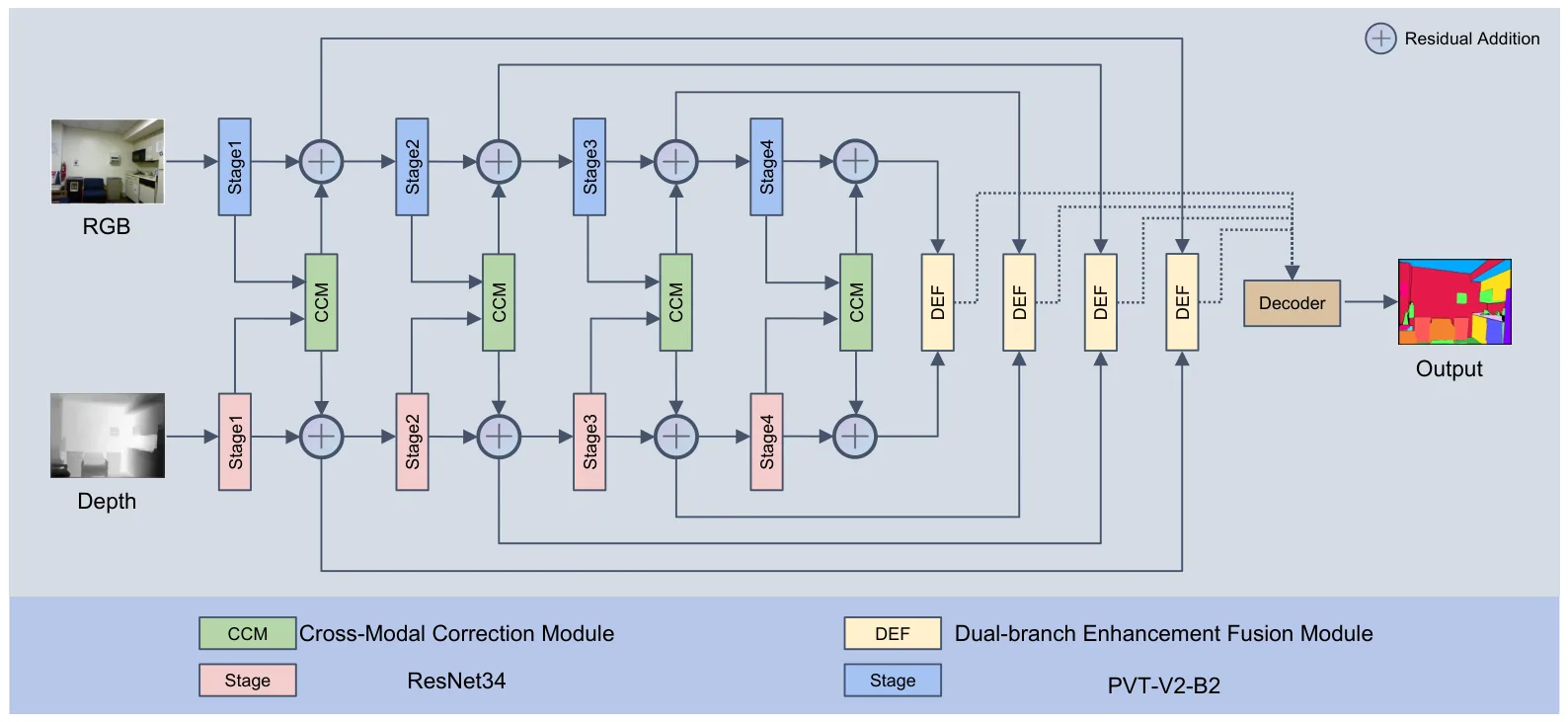
Overall architecture of the Structure-Prior-Guided Network (SPGNet). Red–green–blue (RGB) and depth features are extracted by heterogeneous encoders, corrected by the Cross-Modal Correction Module (CCM) at each stage, enhanced and fused by the Dual-branch Enhancement Fusion Module (DEF), and aggregated by the multi-scale decoder.

As shown in Figure 2, SPGNet contains two encoders, four stage-wise Cross-Modal Correction Modules (CCM), four Dual-branch Enhancement Fusion Modules (DEF), and a multi-scale decoder. At each scale, CCM uses a learned structure prior to regulate cross-modal transfer, after which DEF enhances and fuses the corrected features.

The RGB and depth encoders use Pyramid Vision Transformer v2 B2 (PVT-v2-B2) and 34-layer Residual Network (ResNet34), respectively, with ImageNet-pretrained weights. This pairing combines long-range RGB context with the local geometric features of ResNet34, whose four stages are aligned to PVT-v2-B2 through lightweight projections. For inputs divisible by 32, stage i∈{1,2,3,4} has resolution $\left(h_{i},w_{i}\right)=\left(H/2^{i+1},W/2^{i+1}\right)$ and channels (64,128,320,512), giving

(2) $$F_{r}^{i},F_{d}^{i}\in R^{C_{i}\times h_{i}\times w_{i}},\;\left(C_{1},C_{2},C_{3},C_{4}\right)=\left(64,128,320,512\right).$$

At each stage, the two features are first corrected and then fused:

(3) $$\left({\hat{F}}_{r}^{i},{\hat{F}}_{d}^{i}\right)={\mathrm{CCM}}_{i}\left(F_{r}^{i},F_{d}^{i}\right),$$

(4) $$F_{f}^{i}={\mathrm{DEF}}_{i}\left({\hat{F}}_{r}^{i},{\hat{F}}_{d}^{i}\right),$$

where ${\hat{F}}_{r}^{i}$ and ${\hat{F}}_{d}^{i}$ are the CCM-processed RGB and depth features, and $F_{f}^{i}\in R^{C_{i}\times h_{i}\times w_{i}}$ is the fused feature at stage *i*. The four fused features are aligned to the first-stage resolution and aggregated by the decoder to obtain $\tilde{Y}\in R^{K\times h_{1}\times w_{1}}$:

(5) $$\tilde{Y}=\mathrm{Decoder}\left(F_{f}^{1},F_{f}^{2},F_{f}^{3},F_{f}^{4}\right).$$

The decoder output is then upsampled to the input resolution:

(6) $$Y={\mathrm{Up}}_{\mathrm{ori}}\left(\tilde{Y}\right).$$

Section 3.2 and Section 3.3 describe CCM and DEF, and Section 3.4 specifies the decoder implementation.

### 3.2. Cross-Modal Correction Module

CCM estimates a structure prior before fusion to regulate bidirectional transfer and limit the propagation of unreliable depth responses, boundary inconsistencies, and RGB–depth misalignment.

Given the RGB feature $F_{r}$ and depth feature $F_{d}$ at the same scale, CCM aims to generate the updated bimodal features ${\hat{F}}_{r}$ and ${\hat{F}}_{d}$:

(7) $$\left({\hat{F}}_{r},{\hat{F}}_{d}\right)=\mathrm{CCM}\left(F_{r},F_{d}\right).$$

**Figure 3:**
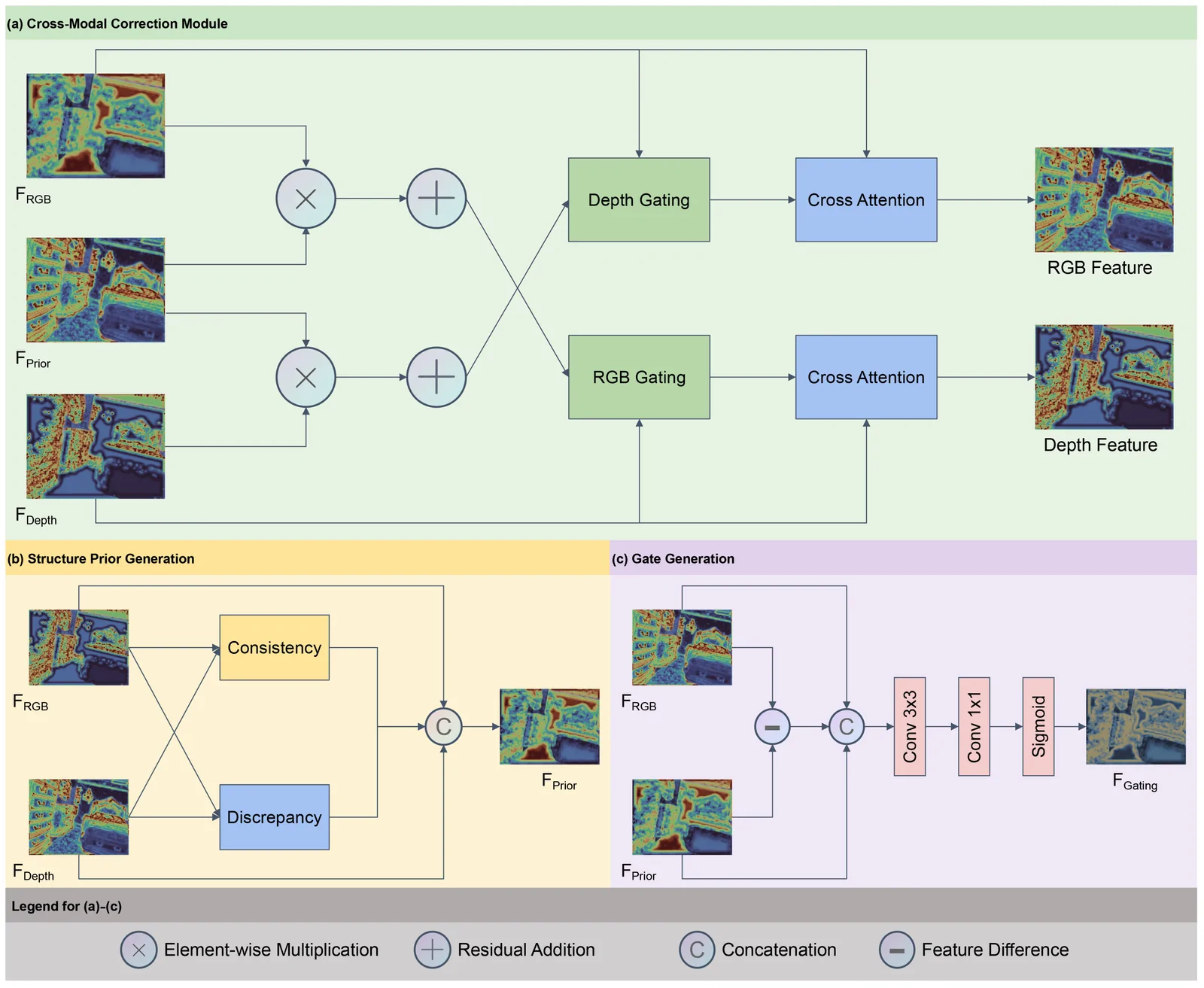
Structure of the Cross-Modal Correction Module (CCM): (**a**) overall architecture; (**b**) fixed-edge and aligned-feature construction of the structure prior; and (**c**) branch-specific gate generation from the target and prior-modulated source features.

As summarized in Figure 3, CCM constructs a prior from cross-modal consistency and discrepancy, generates branch-specific gates, and then performs bidirectional cross-attention.

#### 3.2.1. Structure Prior Generation

Figure 3b maps $F_{r},F_{d}\in R^{C\times h\times w}$ to the structure prior *P* by modeling both cross-modal agreement and discrepancy in the encoded feature space.

The implementation first extracts a fixed structural response from each modality. Let S(F) denote channel averaging followed by convolution with fixed horizontal and vertical Sobel kernels and gradient-magnitude computation. The modality-specific edge responses are

(8) $$E_{r}=S\left(F_{r}\right),\;E_{d}=S\left(F_{d}\right),$$

where $E_{r},E_{d}\in R^{1\times h\times w}$. The edge-consistency response is computed explicitly as

(9) $$C_{s}=1-\frac{|E_{r}-E_{d}|}{E_{r}+E_{d}+\epsilon },$$

with $\epsilon =10^{-6}$. To measure feature discrepancy, the two modalities are independently aligned by bias-free 1×1 convolutions, $A_{r}=\phi_{r}\left(F_{r}\right)$ and $A_{d}=\phi_{d}\left(F_{d}\right)$, and their channel-averaged absolute difference is computed as

(10) $$D_{s}=\frac{1}{C}\sum_{c=1}^{C}\left|A_{r,c}-A_{d,c}\right|.$$

Thus, $C_{s},D_{s}\in R^{1\times h\times w}$. The two edge responses, consistency response, and discrepancy response are concatenated into a four-channel descriptor and mapped to the channel-wise structure prior:

(11) $$P=\sigma \left(F_{p}\left(\left[E_{r},E_{d},C_{s},D_{s}\right]\right)\right),$$

where [·] denotes channel concatenation, $F_{p}\left(\cdot \right)$ comprises two 3×3 Conv–BN–ReLU layers followed by a 1×1 Conv–BN projection, and $P\in {\left[0,1\right]}^{C\times h\times w}$. The consistency and discrepancy quantities are explicit responses; the mapping from their four-channel descriptor to *P* is learned.

#### 3.2.2. Bidirectional Cross-Modal Correction

The prior *P* then guides symmetric depth-to-RGB and RGB-to-depth correction before fusion.

First, the two input feature streams are normalized to obtain

(12) $${\bar{F}}_{r}=\mathrm{BN}\left(F_{r}\right),\;{\bar{F}}_{d}=\mathrm{BN}\left(F_{d}\right).$$

Taking the update of the RGB branch as an example, this paper treats the RGB feature as the target branch and the depth feature as the source branch, and obtains the following representations through 1×1 convolutional mappings:

(13) $$T_{r}=\psi_{r}\left({\bar{F}}_{r}\right),\;S_{d}=\psi_{d}\left({\bar{F}}_{d}\right).$$

Here, $T_{r},S_{d}\in R^{C\times h\times w}$ are the target RGB and source depth representations.

Then, the structure-prior map is used to perform structure-aware modulation on the source-branch feature:

(14) $$S_{d}^{\prime }=S_{d}⊙\left(1+\alpha_{r}P\right),$$

where ⊙ denotes element-wise multiplication, $P\in {\left[0,1\right]}^{C\times h\times w}$ is produced by the Sigmoid output described in the previous subsection, and $\alpha_{r}$ is a learnable scalar initialized to 0. Therefore, this modulation term is an identity mapping at the beginning of training.

Because $\alpha_{r}$ is unbounded, explicit attenuation is performed by the following Sigmoid gate rather than by this modulation term.

Next, the target feature, the prior-modulated source feature, and their difference are concatenated and fed into a gating unit to generate a cross-modal gating map, following the gate-generation pattern in Figure 3c:

(15) $$G_{r}=\sigma \left(F_{g}^{r}\left(\left[T_{r},S_{d}^{\prime },T_{r}-S_{d}^{\prime }\right]\right)\right),$$

where σ(·) denotes the Sigmoid function, and $G_{r}\in {\left[0,1\right]}^{C\times h\times w}$.

The Sigmoid gate attenuates or preserves the modulated source response; the same construction is used in the reverse direction.

After gating, the filtered cross-modal feature is obtained as

(16) $${\tilde{S}}_{d}=S_{d}^{\prime }⊙G_{r}.$$

Thus, CCM filters the source response before cross-modal transfer.

Then, lightweight cross-attention is performed by using the RGB feature as the query and the gated depth feature as the key and value. To control the computational cost, average-pooling downsampling is applied only to the key-value branch in the implementation:

(17) $${\tilde{S}}_{d,↓}={\mathrm{AvgPool}}_{r_{i}}\left({\tilde{S}}_{d}\right),$$

where $r_{i}$ denotes the key-value downsampling rate of CCM at the current stage, and ${\tilde{S}}_{d,↓}\in R^{C\times h_{i}^{↓}\times w_{i}^{↓}}$ denotes the pooled key-value feature with spatial size determined by $r_{i}$. Then,

(18) $$\Delta F_{r}={\mathrm{CA}}_{r}\left(Q=T_{r},K={\tilde{S}}_{d,↓},V={\tilde{S}}_{d,↓}\right).$$

Specifically, ${\mathrm{CA}}_{r}\left(\cdot \right)$ generates the query $Q_{r}$ from $T_{r}$ and generates the key and value $K_{d},V_{d}$ from ${\tilde{S}}_{d,↓}$:

(19) $$Q_{r}=W_{q}^{r}T_{r},\;K_{d}=W_{k}^{r}{\tilde{S}}_{d,↓},\;V_{d}=W_{v}^{r}{\tilde{S}}_{d,↓},$$

The 1×1 projections produce $Q_{r}\in R^{N_{i}d_{i}\times h\times w}$ and $K_{d},V_{d}\in R^{N_{i}d_{i}\times h_{i}^{↓}\times w_{i}^{↓}}$. After attention over flattened spatial dimensions, an output projection restores $\Delta F_{r}\in R^{C\times h\times w}$.

The depth update symmetrically treats depth as the target and RGB as the source:

(20) $$T_{d}=\psi_{d}^{\prime }\left({\bar{F}}_{d}\right),\;S_{r}=\psi_{r}^{\prime }\left({\bar{F}}_{r}\right),$$

(21) $$S_{r}^{\prime }=S_{r}⊙\left(1+\alpha_{d}P\right),$$

(22) $$G_{d}=\sigma \left(F_{g}^{d}\left(\left[T_{d},S_{r}^{\prime },T_{d}-S_{r}^{\prime }\right]\right)\right),$$

(23) $${\tilde{S}}_{r}=S_{r}^{\prime }⊙G_{d},$$

(24) $${\tilde{S}}_{r,↓}={\mathrm{AvgPool}}_{r_{i}}\left({\tilde{S}}_{r}\right),$$

(25) $$\Delta F_{d}={\mathrm{CA}}_{d}\left(Q=T_{d},K={\tilde{S}}_{r,↓},V={\tilde{S}}_{r,↓}\right).$$

Here, $T_{d},S_{r}\in R^{C\times h\times w}$, $G_{d}\in {\left[0,1\right]}^{C\times h\times w}$, and $\alpha_{d}$ is initialized to 0. The projection dimensions match the RGB update: $Q_{d}\in R^{N_{i}d_{i}\times h\times w}$, $K_{r},V_{r}\in R^{N_{i}d_{i}\times h_{i}^{↓}\times w_{i}^{↓}}$, and $\Delta F_{d}\in R^{C\times h\times w}$.

Finally, the bimodal features are updated in a residual manner:

(26) $${\hat{F}}_{r}=F_{r}+\gamma_{r}\Delta F_{r},\;{\hat{F}}_{d}=F_{d}+\gamma_{d}\Delta F_{d},$$

where $\gamma_{r}$ and $\gamma_{d}$ are learnable residual coefficients, both initialized to 0.

The zero initialization of $\alpha_{r}$ and $\alpha_{d}$ makes the prior modulation initially an identity operation, while the zero initialization of $\gamma_{r}$ and $\gamma_{d}$ guarantees that the complete CCM initially preserves its two input features. The residual scales then learn how strongly the cross-modal corrections should be introduced before DEF.

### 3.3. Dual-Branch Enhancement Fusion Module

**Figure 4:**
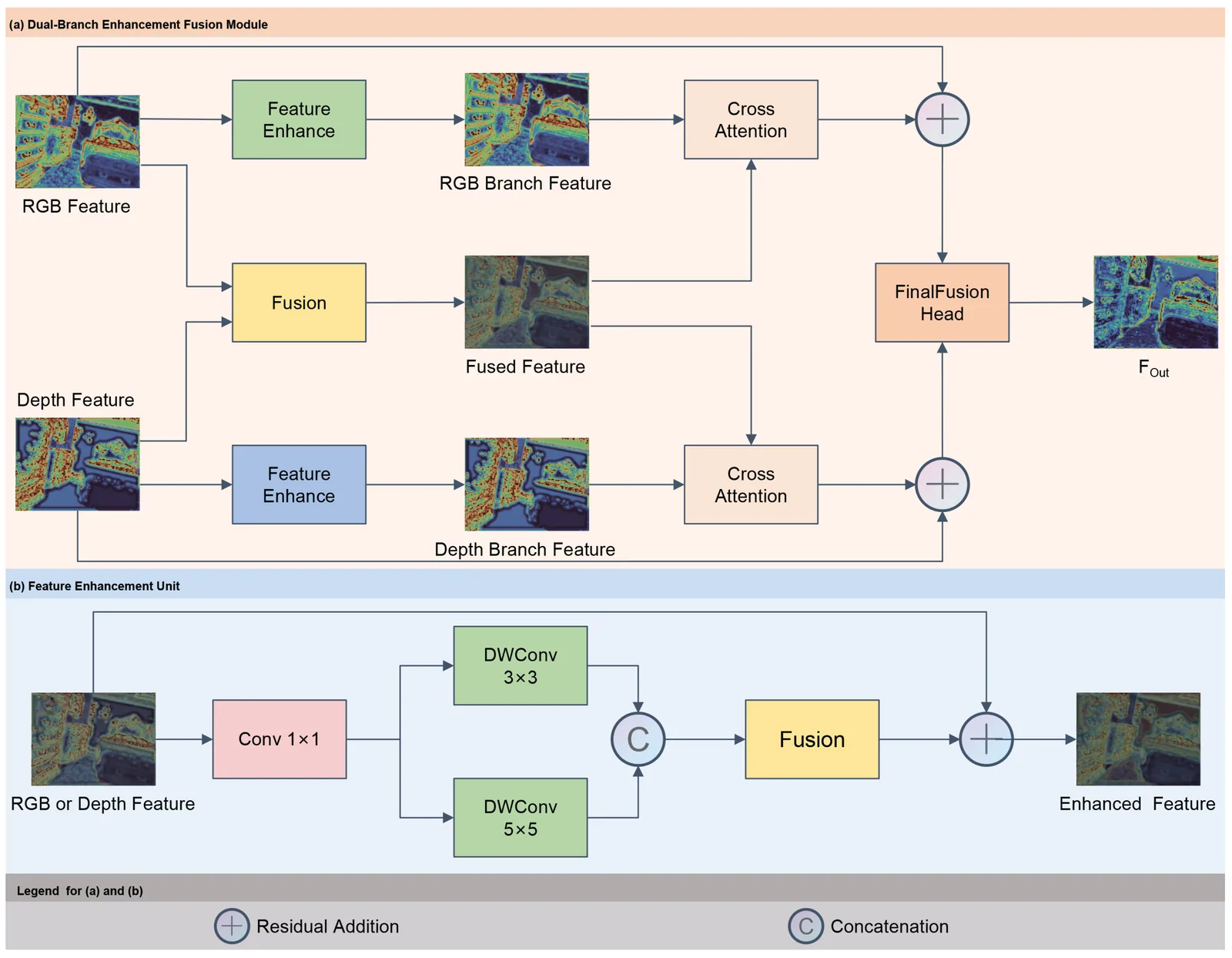
Structure of the Dual-branch Enhancement Fusion Module (DEF): (**a**) overall architecture and (**b**) Feature Enhancement Unit. The operator legend applies to both panels; ⊕ denotes residual addition and *C* denotes channel concatenation.

Given ${\hat{F}}_{r},{\hat{F}}_{d}\in R^{C\times h\times w}$, DEF enhances each modality, constructs a shared reference, applies shared-guided cross-attention, and outputs $F_{f}\in R^{C\times h\times w}$ for the decoder (Figure 4).

#### 3.3.1. Modality Feature Enhancement

As shown in Figure 4b, DEF first enhances the RGB and depth features independently to preserve their modality-specific characteristics. For an arbitrary input feature $F\in R^{C\times h\times w}$, the feature enhancement unit can be expressed as

(27) $$E\left(F\right)=F_{\mathrm{enh}}\left([D_{3\times 3}\left(P\left(F\right)\right),D_{5\times 5}\left(P\left(F\right)\right)]\right)+F,$$

Here, P is a 1×1 projection, $D_{3\times 3}$ and $D_{5\times 5}$ are *C*-channel depthwise separable convolutions, and $F_{\mathrm{enh}}:R^{2C\times h\times w}\to R^{C\times h\times w}$ fuses their concatenated outputs before residual addition.

The two kernels capture complementary local contexts while the residual path preserves the input.

Therefore, the corrected RGB and depth features are enhanced as

(28) $$F_{r}^{\mathrm{enh}}=E\left({\hat{F}}_{r}\right),\;F_{d}^{\mathrm{enh}}=E\left({\hat{F}}_{d}\right).$$

#### 3.3.2. Shared-Guided Cross-Modal Interaction

A shared reference is constructed directly from the corrected bimodal features:

(29) $$F_{m}=F_{m}\left(\left[{\hat{F}}_{r},{\hat{F}}_{d}\right]\right),$$

where $F_{m}\left(\cdot \right):R^{2C\times h\times w}\to R^{C\times h\times w}$ is implemented by a convolutional fusion block, and therefore $F_{m}\in R^{C\times h\times w}$.

Thus, $F_{m}$ retains CCM-corrected shared cues, whereas $F_{r}^{\mathrm{enh}}$ and $F_{d}^{\mathrm{enh}}$ retain modality-specific responses.

Subsequently, convolutional projections are applied to the enhanced RGB feature, the enhanced depth feature, and the shared fused feature, respectively, and all three outputs are kept in $R^{C\times h\times w}$:

(30) $$R=R_{r}\left(F_{r}^{\mathrm{enh}}\right),\;D=R_{d}\left(F_{d}^{\mathrm{enh}}\right),\;M=R_{m}\left(F_{m}\right).$$

Next, two-branch cross-attention interaction is performed by using the RGB-branch feature and the depth-branch feature as queries, respectively, and the shared fused feature as the key and value:

(31) $$R^{\mathrm{ca}}={\mathrm{SGCA}}_{r}^{\mathrm{def}}\left(Q=R,\;K=M_{↓},\;V=M_{↓}\right)+R,$$

(32) $$D^{\mathrm{ca}}={\mathrm{SGCA}}_{d}^{\mathrm{def}}\left(Q=D,\;K=M_{↓},\;V=M_{↓}\right)+D,$$

Only the shared key/value feature is average-pooled: $M_{↓}\in R^{C\times h_{i}^{↓}\times w_{i}^{↓}}$ is determined by $r_{i}^{\prime }$, while the *R* and *D* queries retain resolution h×w. The Q/K/V projections use $16N_{i}$ channels, scaled dot-product attention aggregates *V*, and an output projection restores *C* channels, giving $R^{\mathrm{ca}},D^{\mathrm{ca}}\in R^{C\times h\times w}$.

Using *M* as the common key/value reference aligns both branches without direct RGB–depth attention.

To provide an additional bypass from the corrected DEF inputs, this paper adds the interaction results to the input features of DEF in a residual manner:

(33) $$R^{\prime }=R^{\mathrm{ca}}+{\hat{F}}_{r},\;D^{\prime }=D^{\mathrm{ca}}+{\hat{F}}_{d}.$$

The query-side residuals in the attention equations and the additional corrected-feature bypasses preserve modality information.

Finally, the output feature at the current scale is obtained through the final fusion head, corresponding to the “FinalFusion Head” in Figure 4a:

(34) $$F_{f}=F_{\mathrm{final}}\left(\left[R^{\prime },D^{\prime }\right]\right),$$

where the convolutional head $F_{\mathrm{final}}:R^{2C\times h\times w}\to R^{C\times h\times w}$ produces the decoder input.

### 3.4. Decoder and Multi-Scale Fusion

The decoder integrates $\left\{F_{f}^{1},F_{f}^{2},F_{f}^{3},F_{f}^{4}\right\}$, where $F_{f}^{i}\in R^{C_{i}\times h_{i}\times w_{i}}$ and $\left(C_{1},C_{2},C_{3},C_{4}\right)=\left(64,128,320,512\right)$, using one prediction branch without auxiliary supervision.

First, fused features from the second to fourth stages are upsampled to the first-stage resolution and then concatenated with $F_{f}^{1}$ along the channel dimension:

(35) $$F_{ms}=\mathrm{Concat}\left(F_{f}^{1},{\mathrm{Up}}_{2\to 1}\left(F_{f}^{2}\right),{\mathrm{Up}}_{3\to 1}\left(F_{f}^{3}\right),{\mathrm{Up}}_{4\to 1}\left(F_{f}^{4}\right)\right).$$

Here, ${\mathrm{Up}}_{i\to 1}\left(\cdot \right)$ denotes bilinear interpolation that upsamples the feature of the *i*-th stage to $h_{1}\times w_{1}$. Under the stage-resolution relation $h_{i}=h_{1}/2^{i-1}$ and $w_{i}=w_{1}/2^{i-1}$, the corresponding scale factors are 2,4,8 for the second to fourth stages, respectively. Therefore, $F_{ms}\in R^{\left(64+128+320+512\right)\times h_{1}\times w_{1}}=R^{1024\times h_{1}\times w_{1}}$.

Then, a convolutional fusion layer and a classification layer are used to output logits at the first-stage resolution:

(36) $$\tilde{Y}=F_{\mathrm{cls}}\left(F_{\mathrm{fuse}}\left(F_{ms}\right)\right).$$

$F_{\mathrm{fuse}}$ compresses the concatenated features and $F_{\mathrm{cls}}$ produces $\tilde{Y}\in R^{K\times h_{1}\times w_{1}}$.

Finally, the logits are resized to the original input size H×W to obtain the final output:

(37) $$Y={\mathrm{Up}}_{\mathrm{ori}}\left(\tilde{Y}\right),$$

where $Y\in R^{K\times H\times W}$ contains the final logits.

### 3.5. Loss Function

Let *Y* be the output logits and $Y^{gt}\in {\left\{0,1,\ldots ,K-1\right\}}^{H\times W}$ the ground truth, with label 0 ignored. The valid pixels are

(38) $$\Omega_{v}=\left\{p|Y_{p}^{gt}\neq 0\right\}.$$

The overall loss function is defined as

(39) $$L=L_{seg},$$

where $L_{seg}$ denotes the pixel-level cross-entropy loss over valid pixels:

(40) $$L_{seg}=-\frac{1}{|\Omega_{v}|}\sum_{p\in \Omega_{v}}\log\frac{\exp(Y_{Y_{p}^{gt},p})}{\sum_{k=0}^{K-1}\exp\left(Y_{k,p}\right)}.$$

K=41 for NYU Depth V2 and K=38 for SUN RGB-D, corresponding to 40 and 37 valid classes plus the ignored label. The loss is applied only to the final output.

### 3.6. Implementation Specification of CCM and DEF

Table 1 summarizes the four-scale tensor dimensions and hyperparameters for a 480×640 input. Unless stated otherwise, CCM and DEF convolutional blocks use Conv–BN–ReLU, while alignment, Q/K/V, Sigmoid, and output projections follow the listed rows. The coefficients $\alpha_{r},\alpha_{d},\gamma_{r},\gamma_{d}$ are initialized to 0, making CCM an identity mapping at initialization. The prior hidden dimension is $C_{p}=\max\left(\left\lfloor C/2\right\rfloor ,16\right)$; the CCM head dimension is $d_{i}=\max\left(\left\lfloor C_{i}/\left(2N_{i}\right)\right\rfloor ,16\right)$, and the DEF head dimension is 16. Attention projections use $N_{i}d_{i}$ channels in CCM and $16N_{i}$ in DEF before being mapped back to *C* channels.

### 3.7. Datasets and Experimental Settings

Experiments are conducted on two public RGB-D indoor semantic segmentation benchmarks, namely NYU Depth V2 [2] and SUN RGB-D [3]. Both datasets provide aligned RGB images, depth maps, and pixel-level semantic annotations, and cover diverse indoor scenes with different sensor characteristics and depth response patterns.

NYU Depth V2 contains 1449 indoor scene images. Following the standard split, 795 images are used for training and 654 images are used for testing. The dataset is evaluated under the NYU40 setting with 40 valid semantic classes. SUN RGB-D contains 10,335 RGB-D samples collected from different sources, including NYU Depth V2, Berkeley B3DO, and SUN3D. Following the official split, 5285 images are used for training and 5050 images are used for testing. In this study, SUN RGB-D is evaluated using 37 valid semantic classes. For both datasets, label 0 denotes unlabeled or void pixels and is excluded from both loss computation and metric computation.

All experiments were implemented in Python 3.9 using PyTorch 2.8.0 and NumPy 1.26.4 and conducted on a workstation equipped with an NVIDIA GeForce RTX 5070 Ti GPU. The optimization settings for SPGNet were based on published configurations in recent RGB-D semantic segmentation studies and adapted to our unified experimental protocol. We used AdamW with an initial learning rate of $6\times 10^{-5}$ and a weight decay of 0.01, matching the NYU Depth V2 configuration reported for PrimKD [15]. The use of polynomial learning-rate decay with a power of 0.9 and a warm-up phase was informed by AsymFormer [13]; in our protocol, the warm-up period was set to 5 epochs. To maintain a consistent training length across the reproduced experiments, all models were trained for 100 epochs. The batch sizes were set to 2 for NYU Depth V2 and 4 for SUN RGB-D to fit the available GPU memory. Within each dataset, the same optimization settings were used for SPGNet and the four controlled SUN RGB-D ablation configurations.

The stage-wise hyperparameters of CCM and DEF were specified according to the encoder feature dimensions and the computational cost of spatial attention. For encoder channels of {64,128,320,512}, the numbers of attention heads were set to {1,2,4,4}. The key/value downsampling ratios were {8,4,4,2} for CCM and {8,4,2,1} for DEF. Thus, stronger key/value reduction was applied at high-resolution stages to control the attention cost, whereas lower-resolution stages retained more key/value locations. The DEF per-head dimension was fixed at 16 as a compact implementation choice. The same CCM and DEF configuration was used for both datasets.

The RGB image was used as a three-channel appearance input, while the single-channel depth map was expanded to three channels to match the input structure of the depth encoder. During training, random scale resizing, random horizontal flipping, and random cropping were applied. The random scale was sampled from {0.5,0.75,1.0,1.25,1.5,1.75}, after which each sample was cropped or padded to 480×640. During testing, the samples were processed at the same spatial resolution. RGB images were normalized using the ImageNet mean and standard deviation. For the depth modality, the depth map was first scaled by 1/255 and then normalized using mean values of (0.48,0.48,0.48) and standard deviation values of (0.28,0.28,0.28). The network was trained using the standard cross-entropy loss with label 0 ignored, without auxiliary supervision.

The archived metric exports used for the three-run summaries did not retain a common global seed across Python 3.9, NumPy 1.26.4, and PyTorch 2.8.0; consequently, no seed is assigned retrospectively to these reported runs. They are treated as three independent stochastic training runs, and the sample standard deviations summarize their observed run-to-run variability. In SPGNet, the PVT-v2-B2 RGB encoder and ResNet34 depth encoder were initialized through the pretrained weights provided by their  timm  constructors. Newly introduced convolutional, linear, and normalization layers retained the initialization applied by their PyTorch or  timm  constructors, except for the CCM prior-modulation coefficients $\alpha_{r}$, $\alpha_{d}$ and residual coefficients $\gamma_{r}$, $\gamma_{d}$, which were explicitly initialized to zero as described in Section 3.2.

The reproduced comparison methods include ACNet [27], SA-Gate [9], PrimKD [15], SGACNet [37], ShapeConv [11], and AsymFormer [13]. For each dataset, the proposed method and all reproduced baselines were evaluated using the same input resolution, dataset-specific label-processing procedure, training length, and evaluation code.

### 3.8. Evaluation Metrics

This paper evaluates RGB-D semantic segmentation performance using six metrics: mean Intersection over Union (mIoU), mean F1-score, mean precision, mean recall, Overall Accuracy (OA), and Cohen’s Kappa. mIoU measures the overlap between predicted and ground-truth regions at the class level. Mean precision reflects the average correctness of class-specific positive predictions, mean recall reflects the average coverage of ground-truth pixels across semantic classes, and the mean F1-score balances the two quantities at the class level. OA measures the proportion of correctly classified evaluated pixels, while Kappa further accounts for chance agreement between predictions and ground-truth annotations.

During evaluation, the segmentation head produces K=41 logits for NYU Depth V2 (labels 0–40) and K=38 logits for SUN RGB-D (labels 0–37). Pixels with label 0, representing void or padded regions, are removed before confusion-matrix accumulation and are ignored by the cross-entropy loss. For each valid class c∈{1,…,K−1}, IoU, precision, recall, and F1 are computed from the confusion matrix; mIoU, mean precision, mean recall, and mean F1 are uniformly macro-averaged over labels 1–40 for NYU Depth V2 and labels 1–37 for SUN RGB-D. Masks are resized using nearest-neighbor interpolation and undergo the same spatial transformations as their paired RGB and depth inputs. OA and Kappa are computed from the same confusion matrix over the evaluated non-ignored pixels.

NYU Depth V2 and SUN RGB-D contain 40 and 37 valid categories, respectively, under different processed-label protocols. This study uses the complete-class macro averages above as its primary class-balanced comparison and does not claim that SPGNet improves every individual category or that its gains are concentrated in a predefined object-size group. The archived results contain the aggregate metrics reported in Table 2 and Table 3, but not the per-run class-wise confusion matrices or full-resolution predictions required to reconstruct a consistent complete per-class comparison. Moreover, per-class IoU alone does not identify small- versus large-object behavior because the same semantic category can occupy substantially different image areas. A complete class-wise comparison and instance-scale stratification would therefore require renewed inference under a predefined common protocol and are left as limitations of the present evaluation.

Let $M\in R^{K\times K}$ denote the confusion matrix accumulated over the complete test set, where rows correspond to predicted labels and columns correspond to ground-truth labels. For each valid class *c*, the true-positive, false-positive, and false-negative counts are $TP_{c}=M_{cc}$, $FP_{c}=\sum_{j\neq c}M_{cj}$, and $FN_{c}=\sum_{i\neq c}M_{ic}$, respectively. Class-wise precision, recall, and F1 are then computed as

(41) $$P_{c}=\frac{TP_{c}}{TP_{c}+FP_{c}+\epsilon },\;R_{c}=\frac{TP_{c}}{TP_{c}+FN_{c}+\epsilon },\;F1_{c}=\frac{2P_{c}R_{c}}{P_{c}+R_{c}+\epsilon },$$

where $\epsilon =10^{-10}$ prevents division by zero. Consistent with the implementation, mean precision, mean recall, and mean F1 are macro-averaged over the K−1 valid semantic classes:

(42) $$\bar{P}=\frac{1}{K-1}\sum_{c=1}^{K-1}P_{c},\;\bar{R}=\frac{1}{K-1}\sum_{c=1}^{K-1}R_{c},\;\bar{F1}=\frac{1}{K-1}\sum_{c=1}^{K-1}F1_{c}.$$

## 4. Results and Discussion

**Figure 5:**
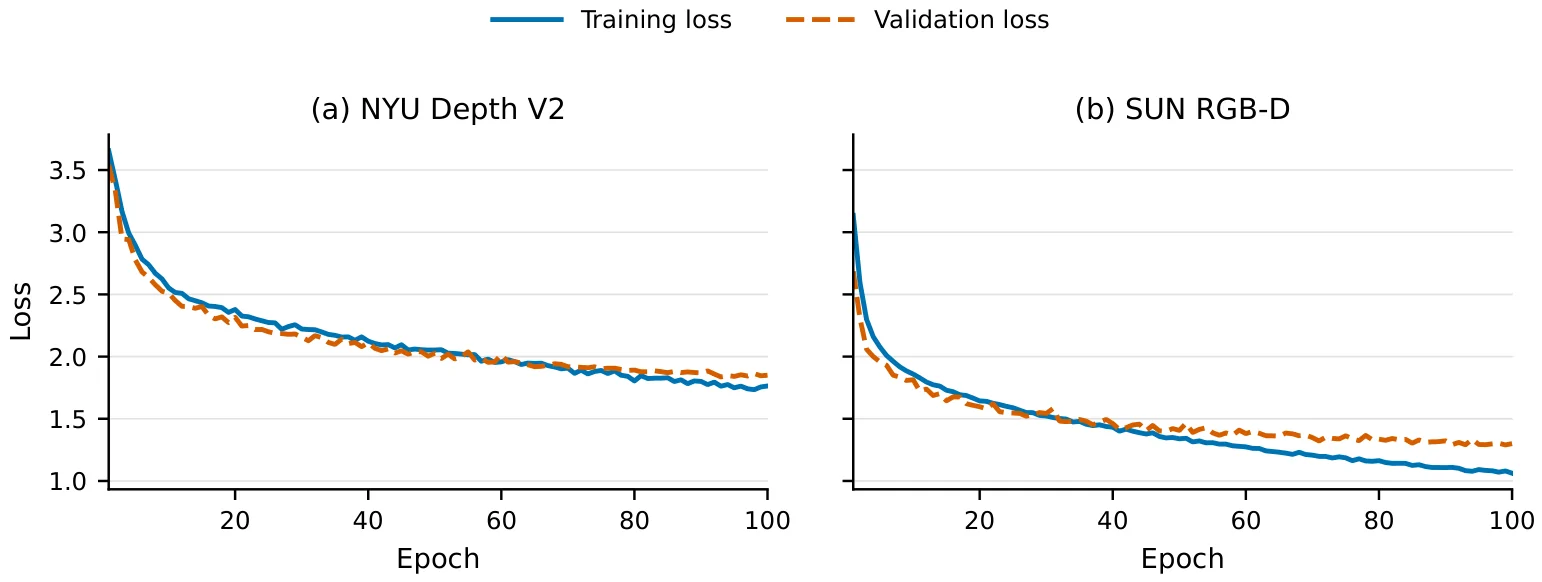
Unsmoothed per-epoch training and validation loss curves of SPGNet over 100 epochs on (**a**) NYU Depth V2 and (**b**) SUN RGB-D.

Figure 5 reports the unsmoothed per-epoch training and validation losses of SPGNet over the complete 100-epoch schedule. On NYU Depth V2, the training loss decreases from 3.656 to 1.764, while the validation loss decreases from 3.540 to 1.851. On SUN RGB-D, the corresponding losses decrease from 3.135 to 1.063 and from 2.686 to 1.300, respectively. For both datasets, the losses decline rapidly during the early epochs and then decrease more gradually, while the validation curves remain stable during the later stage without sustained divergence. These trends indicate stable optimization convergence under the adopted training protocol.

### 4.1. Results on NYU Depth V2

Table 2 presents the quantitative comparison between the proposed method and representative RGB-D semantic segmentation methods on NYU Depth V2 under the unified training and evaluation protocol. Across three independent runs, SPGNet obtains the highest mIoU, F1, mean precision, mean recall, OA, and Kappa values of 50.845±0.126%, 64.985±0.139%, 68.390±1.331%, 64.117±0.326%, 76.497±0.126%, and 73.956±0.073%, respectively. Compared with AsymFormer [13], the second-best method for mIoU and F1, SPGNet improves mean mIoU and mean F1 by 2.111 and 1.681 percentage points. For OA and Kappa, PrimKD [15] gives the second-best three-run means, and SPGNet exceeds it by 0.969 and 1.263 percentage points, respectively.

The gains are also consistent when compared with other convolutional or gating-based RGB-D fusion methods. Using the three-run means, SPGNet improves mIoU over ShapeConv [11], SA-Gate [9], SGACNet [37], and ACNet [27] by 3.635, 3.566, 5.627, and 6.526 percentage points, respectively. These results suggest that applying structure-prior-guided correction before dual-branch enhancement fusion is beneficial under this evaluation setting, while the magnitude of the improvement remains moderate against the strongest transformer-based baseline.

For the additional component metrics, SPGNet achieves the highest three-run mean precision and mean recall of 68.390% and 64.117%, respectively. Compared with AsymFormer, the strongest baseline on these two metrics, SPGNet improves mean precision by 3.149 percentage points and mean recall by 1.350 percentage points. The simultaneous improvements in precision and recall are consistent with the higher mean F1-score obtained by SPGNet on NYU Depth V2.

### 4.2. Results on SUN RGB-D

To further evaluate the proposed method on a larger indoor scene benchmark, this paper reports comparative results on SUN RGB-D. Compared with NYU Depth V2, SUN RGB-D contains more samples from heterogeneous sources and therefore introduces greater variation in scene layout, sensor type, and depth quality. Table 3 summarizes the quantitative results under the corresponding SUN RGB-D processed-label protocol and training pipeline.

Across three independent runs, SPGNet achieves the highest mean mIoU and F1 values of 48.457±0.148% and 62.496±0.076%, respectively. Compared with AsymFormer [13], the second-best method on these metrics, SPGNet improves mean mIoU and mean F1 by 0.899 and 0.963 percentage points. Compared with ShapeConv [11], the corresponding improvements are 3.072 and 2.991 points. Its mean mIoU improvements over PrimKD [15], SA-Gate [9], SGACNet [37], and ACNet [27] are 2.422, 3.345, 4.188, and 4.511 percentage points, respectively. SPGNet also obtains the highest three-run mean OA and Kappa values of 81.183% and 77.900%, exceeding AsymFormer by 0.544 and 0.628 percentage points, respectively.

For the additional component metrics, SPGNet achieves the highest three-run mean precision and mean recall of 64.362% and 61.257%, respectively. Compared with AsymFormer, SPGNet improves mean precision by 0.223 percentage points and mean recall by 1.092 percentage points. These simultaneous gains are consistent with the higher mean F1-score obtained by SPGNet on SUN RGB-D.

To assess statistical significance, two-sided Welch tests were applied to the three independent runs of SPGNet and AsymFormer. On NYU Depth V2, the mIoU difference of 2.111 percentage points has a 95% confidence interval of 1.682–2.540 (p<0.001), and the F1 difference of 1.681 points has a 95% confidence interval of 1.006–2.356 (p=0.005). On SUN RGB-D, the corresponding differences are 0.899 points in mIoU (95% confidence interval: 0.347–1.450; p=0.013) and 0.963 points in F1 (95% confidence interval: 0.237–1.690; p=0.028). Thus, the advantage over the strongest reproduced baseline is retained across the three runs on both datasets. Given the small number of runs, these inferential results are interpreted together with the effect sizes and run-wise variability.

The SUN RGB-D results follow the same trend as the NYU Depth V2 results: SPGNet consistently improves over the reproduced baselines, while the margin over the strongest transformer-based baseline remains relatively small. This observation suggests that the proposed correction-and-fusion design is useful across the two evaluated indoor RGB-D benchmarks, but the results should be interpreted together with the following model-scale analysis.

**Figure 6:**
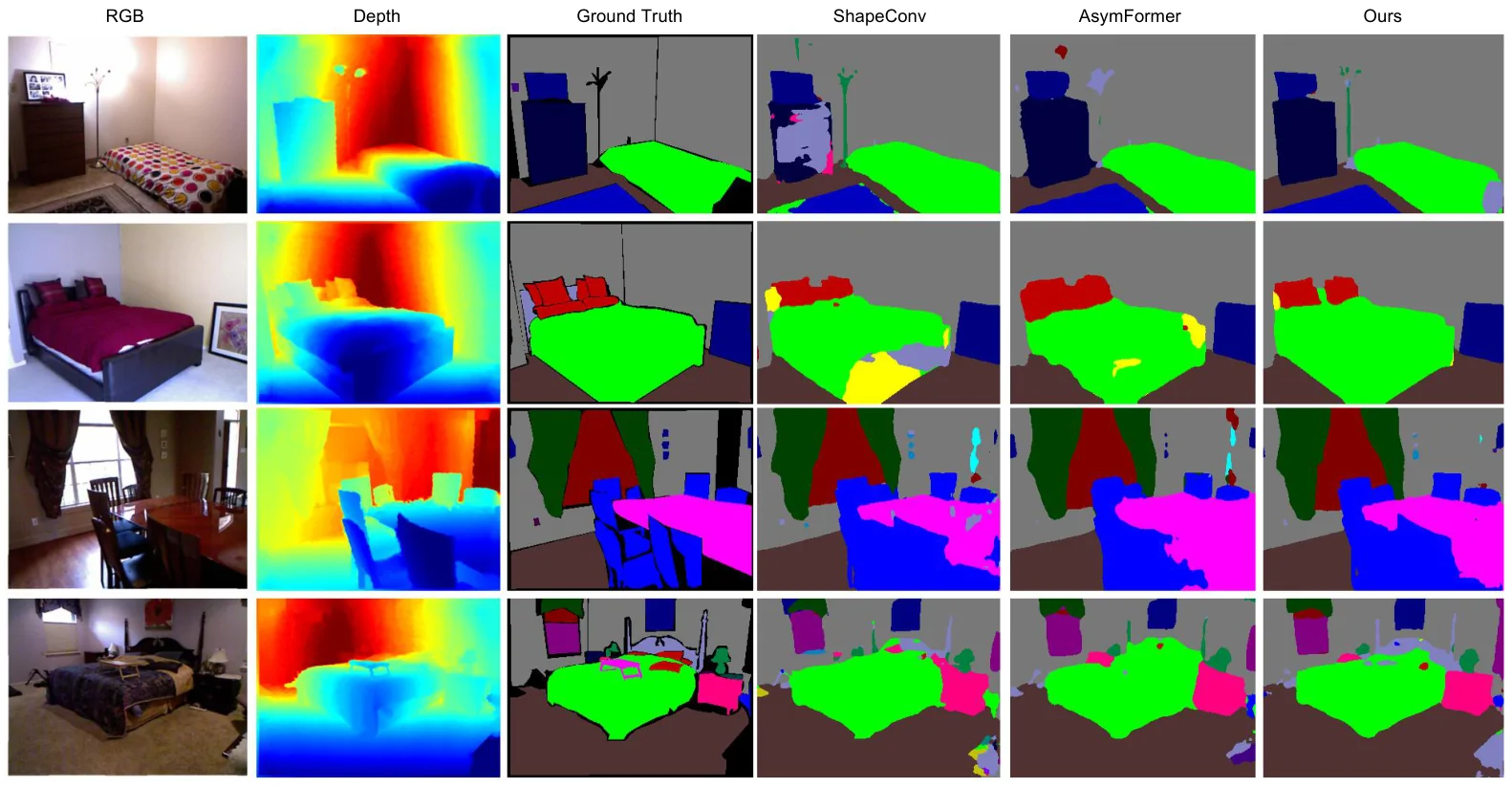
Qualitative comparison on NYU Depth V2. From left to right, the columns show the RGB image, depth map, ground truth, ShapeConv, AsymFormer, and SPGNet predictions.

**Figure 7:**
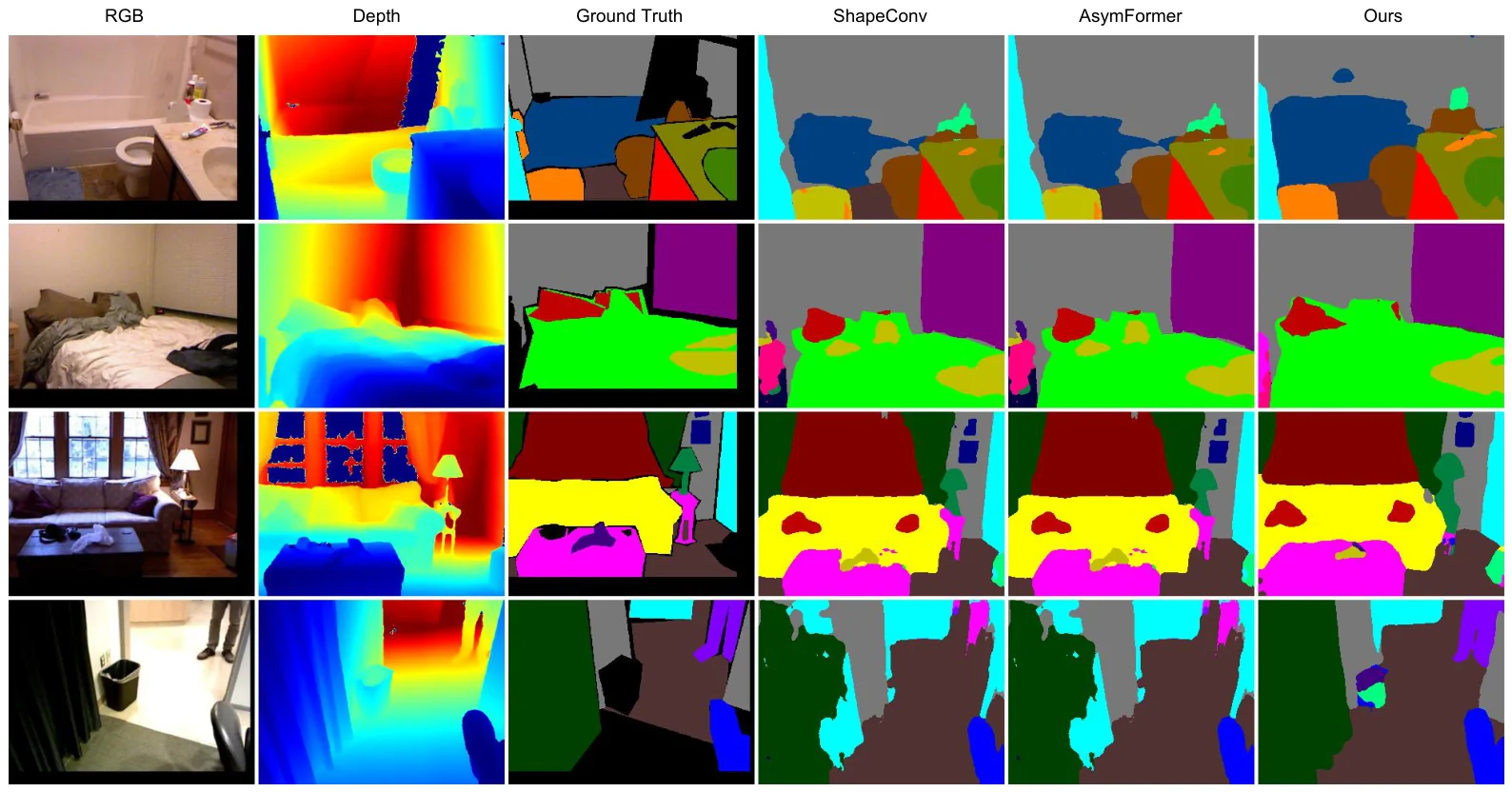
Qualitative comparison on SUN RGB-D. From left to right, the columns show the RGB image, depth map, ground truth, ShapeConv, AsymFormer, and SPGNet predictions.

Figure 6 and Figure 7 present qualitative comparisons on NYU Depth V2 and SUN RGB-D, respectively.

In the first NYU Depth V2 example, ShapeConv produces fragmented labels on the tall cabinet and around the bed–floor transition, while AsymFormer introduces a small isolated false-positive region above the furniture. SPGNet yields a more contiguous cabinet region and a cleaner bed contour against the wall and floor. In the second and fourth examples, the bed occupies a large portion of the scene and is surrounded by pillows, nearby furniture, and weak wall–floor depth transitions. SPGNet preserves a more complete bed region with less label leakage into the surrounding background. In the dining-room example, the proposed model also maintains a coherent tabletop region and separates much of the adjacent furniture, although thin chair structures and small objects remain difficult for all three methods.

A similar pattern is visible on SUN RGB-D. In the bathroom and bedroom examples, SPGNet better preserves the dominant object extent at the bathtub–wall and bed–background interfaces, where the depth maps contain sharp transitions together with locally noisy or weak responses. In the living-room example, the sofa region remains more spatially coherent, whereas the comparison predictions contain more local boundary irregularities. The final doorway scene illustrates a harder case involving narrow foreground structures and partial occlusion: SPGNet recovers more of the small foreground regions but still exhibits errors near ambiguous boundaries. These observations are consistent with the intended roles of CCM and DEF: CCM can reduce the influence of locally inconsistent cross-modal responses before fusion, while DEF can combine the corrected modalities to refine object extent and boundaries. However, the end-to-end visual comparisons alone do not isolate the causal contribution of either module; that evidence is provided by the controlled module ablation in Section 4.4.

**Figure 8:**
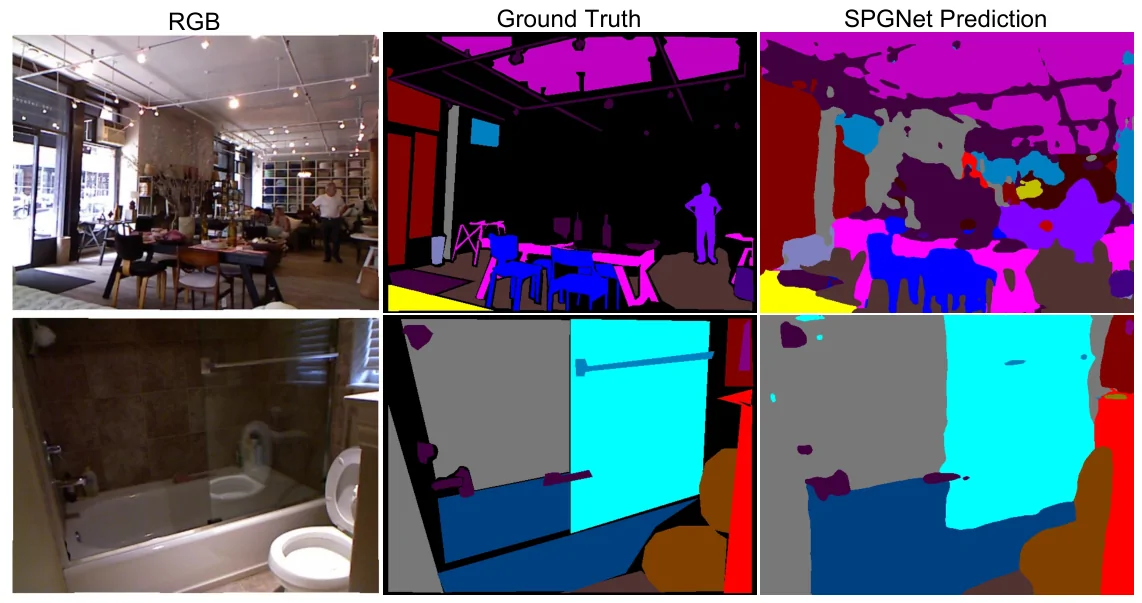
Representative failure cases of SPGNet. From left to right, the columns show the RGB image, ground-truth annotation, and SPGNet prediction. Black pixels in the ground truth correspond to ignore index 0 and are excluded from the loss and quantitative evaluation. The top row illustrates a cluttered scene with extensive ignored regions, dense small objects, and occlusion; the bottom row illustrates boundary displacement and loss of thin structures in a dim bathroom scene.

**Representative Failure Cases.** To complement the qualitative comparisons above, Figure 8 presents two representative failure cases that illustrate recurring error mechanisms under challenging indoor conditions.

The first case contains densely arranged furniture, small objects, and substantial occlusion. A large part of the visually complex background is assigned to the ignored category in the reference annotation, whereas SPGNet predicts semantic labels for several visible scene elements in that area. Because predictions at ignored pixels are excluded from the quantitative metrics, these colored regions should not be interpreted directly as segmentation errors. Within the labeled regions, however, the prediction remains locally fragmented around overlapping tables, chairs, and background furnishings. This example indicates that densely packed small structures, severe occlusion, and ambiguous interfaces can still cause confusion between adjacent object categories, while also demonstrating how incomplete annotations can complicate qualitative interpretation.

In the second case, SPGNet recovers the dominant semantic regions of the bathroom but loses several fine structural details. The predicted cyan region extends irregularly across the upper boundary of the dark-blue bathtub region, producing local boundary displacement and label leakage. Thin structures in the reference annotation, including the narrow fixture and bathtub rim, are omitted or absorbed into adjacent regions, and the object interfaces on the right are oversmoothed. Weak illumination, reflective or transparent surfaces, and thin metallic structures provide ambiguous appearance and geometric cues. When both modalities contain weak or inconsistent boundary evidence, the learned structure prior may therefore fail to preserve narrow structures or regulate cross-modal transfer accurately near object interfaces. These observations are consistent with the limitations discussed in Section 5 and motivate boundary-aware supervision, explicit uncertainty modeling, and broader failure analysis on held-out data.

The boundary observations in Figure 6, Figure 7 and Figure 8 are qualitative and should not be interpreted as a quantitative boundary-specific advantage. Boundary IoU and Boundary F1 depend on the boundary-extraction operator, matching tolerance or band width, treatment of ignored pixels, and normalization with respect to image resolution. These choices are particularly consequential for annotations containing coarse interfaces or extensive ignored regions. The archived evaluations do not retain full-resolution predictions for all reproduced methods; therefore, a controlled boundary comparison would require renewed inference for every method after fixing one common extraction, tolerance, ignored-pixel, and resolution-normalization protocol. This additional experiment is not included in the present revision, which retains the standard benchmark metrics and limits its boundary claims accordingly.

**Figure 9:**
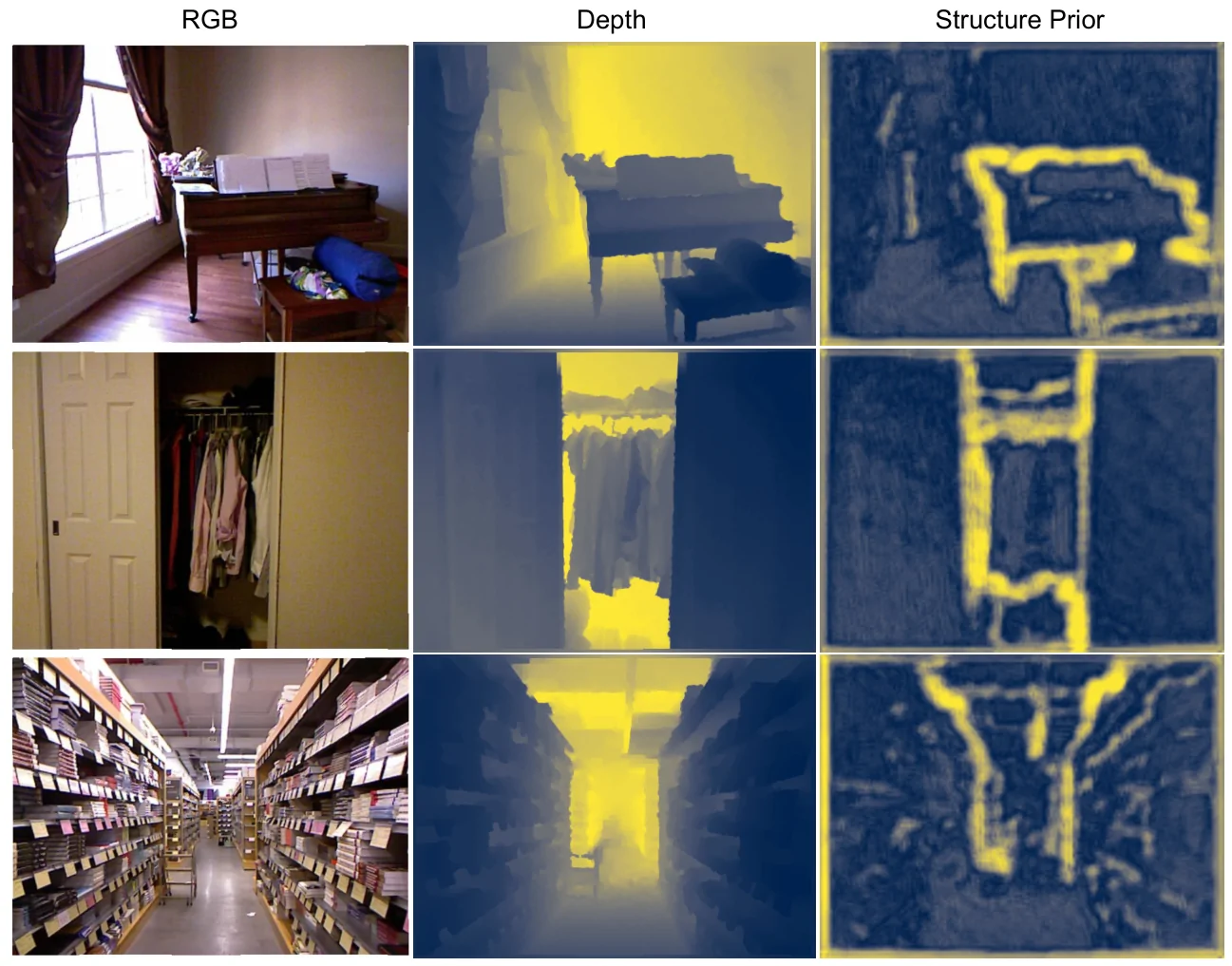
Visualization of learned Stage 1 structure priors generated by the Cross-Modal Correction Module (CCM) on NYU Depth V2. Each row shows the RGB image, the corresponding depth input, and the learned structure-prior response. For visualization, the Stage 1 prior tensor is averaged across channels, bilinearly upsampled to the input resolution, independently linearly scaled between its 1st and 99th percentiles, and shown without spatial smoothing. Brighter colors indicate stronger relative responses within the same map and should not be interpreted as absolute values comparable across samples.

**Visualization of the Learned Structure Prior.** To examine the intermediate representation used by CCM, Figure 9 visualizes representative Stage 1 structure-prior maps on NYU Depth V2.

The displayed response maps exhibit spatially organized patterns around prominent scene structures, including the piano and bench contours in the first example, the closet opening and nearby object boundaries in the second, and the shelf–aisle layout in the third. These responses are not identical to either raw RGB texture or depth intensity; instead, they summarize the learned prior constructed from joint consistency and discrepancy cues in the two encoded modalities. The maps therefore provide qualitative evidence that CCM forms a structure-sensitive intermediate representation before feature fusion. This visualization alone does not prove suppression of unreliable information, but, together with the controlled CCM ablation reported in Section 4.4, it supports the interpretation that the learned prior supplies spatial guidance for regulating subsequent cross-modal transfer.

### 4.3. Robustness Analysis

To isolate robustness to unreliable depth, we additionally evaluated the compared methods on the full NYU Depth V2 test set while keeping the RGB images and ground-truth labels unchanged. Only the normalized depth input was perturbed. Zero-mean Gaussian noise with standard deviations of 0.05, 0.10, and 0.20 was added to the depth tensor. For the misregistration test, only the depth map was translated by 1, 3, or 5 pixels along a cardinal direction selected independently for each test sample, with replicated boundary values. One fixed trained checkpoint per method was evaluated once for every condition; no retraining or test-time adaptation was performed. Table 4 reports mIoU, and the values in parentheses denote the absolute change in percentage points relative to the clean result from the same checkpoint. Because the archived robustness outputs did not retain the random perturbation seed, this table should be interpreted as a single-realization sensitivity analysis rather than a variance estimate over perturbation realizations.

SPGNet retains the highest absolute mIoU under every evaluated depth perturbation. Its mIoU decreases from 50.78% on clean inputs to 50.22%, 49.41%, and 48.64% as the depth-noise level increases, remaining 0.12 percentage points above AsymFormer at σ=0.20. Under depth-only shifts of 1, 3, and 5 pixels, SPGNet obtains 50.79%, 50.78%, and 50.78% mIoU, respectively, and exceeds AsymFormer by 2.15 points at 5 pixels. These absolute results show that SPGNet preserves the strongest segmentation performance across the tested depth corruptions. Nevertheless, AsymFormer exhibits smaller relative decreases under the stronger noise settings; therefore, the evidence supports the highest retained absolute accuracy for SPGNet rather than the smallest degradation in every condition. Large contiguous missing-depth regions remain outside the scope of this experiment.

### 4.4. Ablation Study

**Figure 10:**
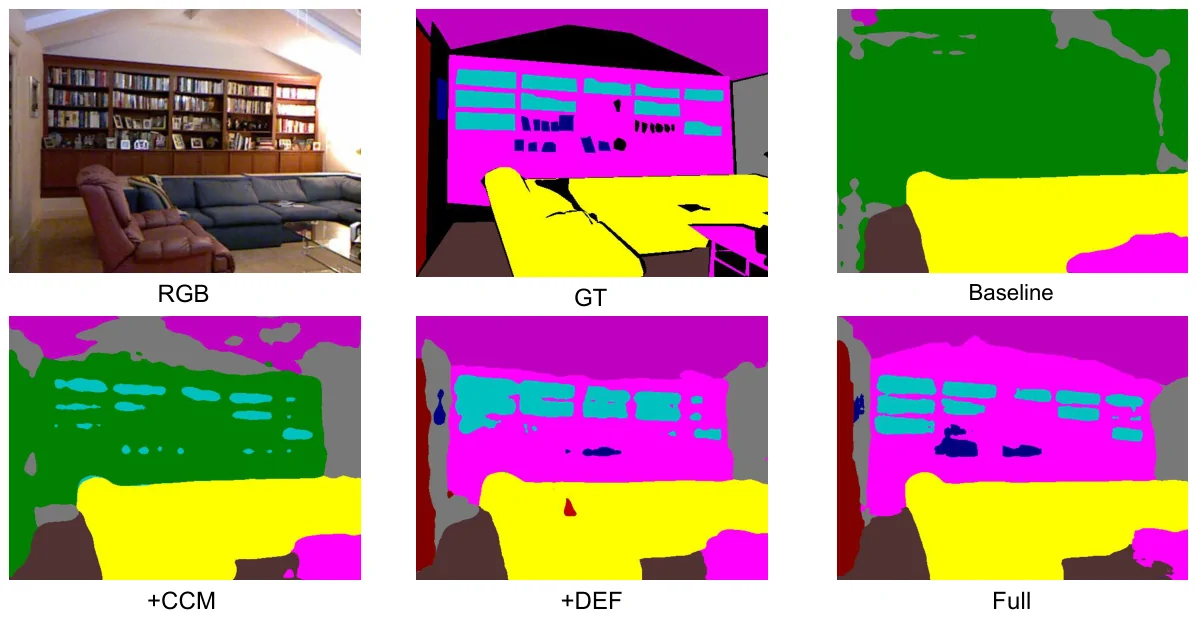
Visual ablation comparison on SUN RGB-D. The panels show the RGB image, ground truth, Baseline, Baseline+CCM, Baseline+DEF, and the full SPGNet prediction.

To evaluate the contribution of each key component, this paper conducts step-by-step ablation experiments on SUN RGB-D under the unified training setting. All ablation settings use the same heterogeneous dual-branch encoder and differ only in whether CCM and DEF are enabled. The compared settings include *Baseline*, which uses neither CCM nor DEF; *Baseline+CCM*, which introduces only the cross-modal correction module; *Baseline+DEF*, which introduces only the dual-branch enhancement fusion module; and *Full*, which uses both CCM and DEF. In particular, Baseline+DEF is the fusion-only configuration: DEF remains enabled while CCM is removed, so the dual-modal features are enhanced and fused without preceding structure-prior-guided correction. The PVT-v2-B2 RGB encoder, ResNet34 depth encoder, multi-scale decoder, training schedule, and evaluation protocol are kept fixed across all four configurations. Therefore, the observed performance differences isolate the contributions of CCM and DEF rather than changes in the feature extractors. The quantitative results are reported in Table 5, and a representative visual comparison is shown in Figure 10.

Adding CCM alone improves mIoU from 45.395% to 46.375% (+0.980) and F1 from 59.309% to 60.271% (+0.962). Adding DEF alone improves mIoU to 45.817% (+0.422) and F1 to 59.651% (+0.342), although its OA and Kappa are slightly lower than those of the Baseline. Thus, DEF alone mainly improves overlap- and F1-related metrics on SUN RGB-D, while its effect on pixel-level accuracy is weaker when CCM is absent.

With both modules enabled, the full model achieves 48.418% mIoU, 62.418% F1, 81.217% OA, and 77.953% Kappa. Compared with the Baseline, the gains are 3.023, 3.109, 1.468, and 1.714 percentage points, respectively. More directly, comparing the full model with the fusion-only Baseline+DEF configuration measures the incremental benefit of adding CCM before DEF under otherwise identical settings. Adding pre-fusion correction increases mIoU from 45.817% to 48.418% (+2.601), F1 from 59.651% to 62.418% (+2.767), OA from 79.483% to 81.217% (+1.734), and Kappa from 76.009% to 77.953% (+1.944). On SUN RGB-D under the unified protocol, this controlled comparison shows that applying CCM before DEF improves final segmentation performance relative to the fusion-only configuration within the evaluated architecture. The full model outperforms both single-module variants on all four metrics, indicating that cross-modal correction and dual-branch enhancement fusion provide complementary contributions when used jointly. The visual example in Figure 10 further illustrates that the full model produces more complete object regions than the Baseline in this SUN RGB-D scene.

### 4.5. Model Complexity Analysis

To better characterize the trade-off between accuracy and model scale across different methods, this section re-profiles the RGB-D semantic segmentation methods appearing in the main quantitative comparisons using the local profiling framework, as shown in Table 6. All models are evaluated in inference mode with batch size 1 at 480×640, using three-channel RGB images and one-channel depth maps; the depth map is repeated internally only when a model interface requires three channels. Parameter counts are computed directly from the instantiated models, and computational costs are reported as FLOPs under the common convention that one multiply–accumulate operation corresponds to two floating-point operations. For SPGNet, no layer is frozen during training and all model parameters are passed to the AdamW optimizer; therefore, its reported parameter count is also its trainable parameter count. End-to-end latency and throughput for every method are measured on an NVIDIA GeForce RTX 5070 Ti GPU under this common input protocol. CUDA synchronization is applied before and after each timed measurement, and timing begins only after 50 warm-up iterations. For each method, the reported latency is averaged over 1500 timed forward passes from three independent measurement runs, and frames per second (FPS) is computed as the reciprocal of the mean per-image latency. Timing covers the model forward pass to full-resolution logits and excludes model construction and host-to-device transfer. Peak allocated GPU memory is measured after warm-up by resetting the CUDA peak-memory statistics for each independent run, and the three run-wise peaks are averaged. Since fvcore does not support every operator used by these models, the FLOP values are partial profiling results and are intended for model-scale reference rather than exact hardware-independent complexity comparison. The profiled backbone column reflects the local adapter instantiation used for complexity measurement, while the mIoU columns follow the main quantitative comparison tables.

In terms of parameter count, SPGNet contains a heterogeneous encoder, four-stage CCM, four-stage DEF, and a multi-scale fusion decoder, resulting in 122.21 M trainable parameters. This places it in the relatively large-model group among the locally instantiated models in this table. Its parameter count is higher than those of ACNet, SA-Gate, SGACNet, ShapeConv, and AsymFormer, but lower than that of PrimKD with MiT-B4 under the current local profiling setup.

In terms of computational cost, SPGNet requires 99.91 G partially profiled FLOPs under the local setup. Because unsupported operators differ across architectures, these FLOP values are provided as implementation-specific diagnostics and are not used for strict cross-model ranking. The common runtime measurements show that SPGNet requires 34.64 ms per image (28.87 FPS). It is slower than ACNet, SA-Gate, SGACNet, ShapeConv, and AsymFormer, but faster than PrimKD under the tested hardware and implementation environment. These results provide a hardware-specific indication of practical deployment cost. Combined with the accuracy results in Table 2 and Table 3, SPGNet achieves the highest mIoU on both NYU Depth V2 and SUN RGB-D among the compared methods, while requiring a relatively large parameter count and mid-range measured latency. Therefore, the proposed method should be regarded as an accuracy-oriented model rather than a lightweight design. Future work may further explore lightweight attention projections and channel transformations in CCM and DEF.

To isolate the computational overhead associated with CCM and DEF, the four controlled ablation configurations are also profiled under the same hardware, input resolution, batch size, inference mode, and timing protocol. As reported in Table 7, Baseline+CCM and Baseline+DEF differ from the common Baseline only by the corresponding module, while the full SPGNet enables both modules. This controlled profiling separates module-related changes in parameter count, FLOPs, latency, and peak allocated GPU memory from changes in the encoders, decoder, or input protocol. Runtime and peak-memory values are interpreted per configuration rather than added across rows because kernel execution and memory reuse are not strictly additive.

## 5. Conclusions

This paper proposes the Structure-Prior-Guided Network (SPGNet) for RGB-D semantic segmentation, with the aim of improving multimodal feature interaction under cross-modal response inconsistency, unreliable depth-response propagation, and limited multi-scale fusion. SPGNet adopts a heterogeneous dual-encoder architecture to model RGB appearance semantics and depth geometric structures separately, and applies the Cross-Modal Correction Module (CCM) and the Dual-branch Enhancement Fusion Module (DEF) sequentially at four scales. CCM constructs structure priors from RGB–depth consistency and discrepancy cues, and uses these priors for structural modulation, gated selection, and bidirectional cross-modal correction before fusion. DEF further enhances the corrected dual-branch features and performs shared-guided interaction before the multi-scale fusion decoder aggregates features from different levels for pixel-level prediction.

Under the dataset-specific processed-label protocols used in this paper, SPGNet obtains three-run mean mIoU, F1, OA, and Kappa values of 50.845%, 64.985%, 76.497%, and 73.956% on NYU Depth V2 and 48.457%, 62.496%, 81.183%, and 77.900% on SUN RGB-D, respectively. These results are favorable among the compared representative RGB-D segmentation methods in the reported evaluation setting. The ablation experiments further show that, under the same heterogeneous dual-branch encoder, jointly enabling CCM and DEF improves all four metrics on SUN RGB-D, which is consistent with the complementary roles of pre-fusion structural correction and subsequent enhancement fusion. The complexity analysis indicates that these accuracy gains are accompanied by a relatively large parameter count and computational cost, positioning SPGNet as an accuracy-oriented model rather than a lightweight design.

This study has several limitations. First, although the added depth-only Gaussian-noise and spatial-shift experiments evaluate SPGNet under controlled depth corruption while preserving the RGB input, they do not cover large contiguous missing-depth regions. The effects of severe depth dropout and more substantial RGB–depth misregistration therefore require further study. Second, the current experiments are limited to indoor RGB-D datasets; therefore, generalization to outdoor scenes with different sensing ranges, illumination conditions, depth statistics, and class distributions remains unverified. Transferability to other spatially aligned multimodal segmentation settings, such as RGB–thermal or RGB–LiDAR segmentation, also remains to be established. Because CCM and DEF operate on paired stage-wise feature maps rather than raw sensor-specific values, the framework could in principle be adapted by replacing the depth encoder and relearning modality-specific structure priors. However, differences in sensor alignment, noise characteristics, and spatial sparsity require task-specific validation. Third, SPGNet is an accuracy-oriented rather than lightweight model. It contains 122.21 M trainable parameters, and Table 6 reports 99.91 G FLOPs for the profiled portion of the model at an input resolution of 480×640. The parameter count is independent of input resolution, whereas the computational and memory costs increase as the resolution grows. For convolutional components with fixed channel dimensions and kernels, the computational cost grows approximately linearly with the number of spatial locations. In CCM and DEF, the query retains the full spatial resolution at each stage, while the key and value maps are average-pooled by a ratio *r* along both spatial dimensions. Thus, for a stage feature map with $N=h_{i}w_{i}$ query locations, the key/value sequence contains approximately $N/r^{2}$ locations, and the cross-attention score computation and score-matrix memory scale as $O(N^{2}/r^{2})$, assuming fixed channel dimensions. Consequently, if the input height and width are both increased by a factor of *s*, convolutional cost grows approximately as $s^{2}$, whereas this attention term grows approximately as $s^{4}$ for a fixed *r*. The stage-wise key/value downsampling ratios used in CCM ({8,4,4,2}) and DEF ({8,4,2,1}) reduce, but do not eliminate, this resolution-dependent growth. Finally, scenes involving severe occlusion, weak or ambiguous boundaries, reflective or transparent surfaces, or strong RGB–depth misalignment may remain challenging. The diagnostic examples in Figure 8 concretely illustrate fragmentation under clutter and occlusion, as well as boundary displacement and loss of thin structures under weak and reflective visual conditions. Although controlled depth noise and shift perturbations are now evaluated, these individual scene and sensor failure modes still require more systematic investigation on held-out data. Complete per-class behavior, instance-scale-stratified performance, and boundary-focused accuracy under a predefined extraction and tolerance protocol also remain to be evaluated.

Future work will focus on three directions. First, the parameter overhead introduced by attention projections and multi-channel convolutions in CCM and DEF can be reduced to improve deployment feasibility in real-time or resource-constrained scenarios. This direction will include evaluating lightweight convolutional networks and hierarchical Transformer architectures as alternative depth encoders to characterize the accuracy–efficiency trade-off and the generality of the heterogeneous encoder design. Second, robustness to controlled levels of depth degradation and the generalization ability of structure-prior-guided correction can be evaluated on more indoor and outdoor RGB-D datasets, cross-domain settings, and other spatially aligned multimodal segmentation tasks. Finally, more fine-grained visualization and error-case analyses can be incorporated to better explain the roles of structure priors, gated selection, and enhancement fusion for different scene categories and boundary regions. This direction will use a predefined protocol to report complete per-class results, stratify performance by instance scale and boundary difficulty, and quantify boundary accuracy under explicitly stated extraction operators and tolerance widths.

## Figures and Tables

**Table 1:** Notation, dimensions, and hyper-parameter specifications of the Cross-Modal Correction Module (CCM) and Dual-branch Enhancement Fusion Module (DEF) used in this work. Subscripts *r* and *d* denote the red–green–blue (RGB) and depth branches, respectively. C∈{64,128,320,512} denotes the output channels of stages 1–4; h×w denotes the corresponding spatial resolution; $N_{i}$ denotes the number of attention heads, $d_{i}$ denotes the CCM channels per head, d=16 denotes the fixed DEF channels per head, and $r_{i}/r_{i}^{\prime }$ denote the key/value downsampling ratios in cross-attention.

Symbol	Shape/Setting	Description
*Inputs and stage features*		
$I_{r},{\tilde{I}}_{d}$	3×H×W	RGB/channel-expanded depth input
$F_{r}^{i},F_{d}^{i}$	$C_{i}\times h_{i}\times w_{i}$	Stage-*i* encoder outputs (RGB/depth)
${\hat{F}}_{r}^{i},{\hat{F}}_{d}^{i}$	$C_{i}\times h_{i}\times w_{i}$	CCM-corrected features
$F_{f}^{i}$	$C_{i}\times h_{i}\times w_{i}$	DEF-fused features at stage *i*
*Structure prior generation*		
$E_{r},E_{d}$	1×h×w	Fixed Sobel edge magnitudes of channel-averaged RGB/depth features
$\phi_{r},\phi_{d}$	C→C	Independent 1×1 convolutional alignment for discrepancy computation
$A_{r},A_{d}$	C×h×w	Aligned RGB/depth features
$C_{s},D_{s}$	1×h×w	Edge-consistency and channel-averaged feature-discrepancy responses
$C_{p}$	max(⌊C/2⌋,16)	Hidden channels of prior generator
$F_{p}$	$4\to C_{p}\to C_{p}\to C$	Two 3×3 Conv–BN–ReLU layers + 1×1 Conv–BN before Sigmoid
*P*	C×h×w	Sigmoid-bounded structure prior
*CCM (per branch and per stage)*		
${\bar{F}}_{r/d}$	C×h×w	BN-normalized features
$\psi_{r},\psi_{d},\psi_{r}^{\prime },\psi_{d}^{\prime }$	C→C	1×1 Conv–BN–ReLU target/source projection
$\alpha_{r},\alpha_{d}$	scalar	Learnable, unconstrained-sign, init=0
$C_{g}$	max(⌊C/2⌋,32)	Hidden channels of branch-specific gate generator
$F_{g}^{r/d}$	$3C\to C_{g}\to C$	Target/prior-modulated source/difference input; 3×3 Conv–BN–ReLU + 1×1 Conv before Sigmoid
$G_{r},G_{d}$	C×h×w	Cross-modal gating mask, ∈[0,1]
${\tilde{S}}_{r/d,↓}$	$C\times \left\lfloor \frac{h}{r_{i}}\right\rfloor \times \left\lfloor \frac{w}{r_{i}}\right\rfloor$	Avg-pooled source KV feature in CCM
${\mathrm{CA}}_{r},{\mathrm{CA}}_{d}$	$N_{i},d_{i}=\max\left(\left\lfloor C_{i}/\left(2N_{i}\right)\right\rfloor ,16\right),r=r_{i}$	Multi-head cross-attention; Q/K/V dim $=N_{i}d_{i}$; output projection $\to C_{i}$
$\gamma_{r},\gamma_{d}$	scalar	Learnable residual scale, init=0
*DEF (per stage)*		
E(·)	C→C	1×1 pre + DW(3×3/5×5) + conv fusion block
$F_{r}^{\mathrm{enh}},F_{d}^{\mathrm{enh}}$	C×h×w	Modality-enhanced features
$R_{r},R_{d}$	C→C	3×3 Conv–BN–ReLU projection of enhanced features
$F_{m}$	2C→C	Conv fusion block for shared feature construction
$F_{m}$	C×h×w	Shared fusion feature
$R_{m}$	C→C	3×3 Conv–BN–ReLU projection of shared feature
$M_{↓}$	$C\times \left\lfloor \frac{h}{r_{i}^{\prime }}\right\rfloor \times \left\lfloor \frac{w}{r_{i}^{\prime }}\right\rfloor$	Avg-pooled shared KV feature in DEF
${\mathrm{SGCA}}_{r}^{\mathrm{def}},{\mathrm{SGCA}}_{d}^{\mathrm{def}}$	$N_{i},d=16,r=r_{i}^{\prime }$	Shared-guided cross-attention; Q/K/V dim $=N_{i}d$; output projection $\to C_{i}$; shared KV
$F_{\mathrm{final}}$	2C→C	Conv fusion head (1×1 Conv–BN–ReLU + 3×3 Conv–BN–ReLU + 1×1 Conv–BN–ReLU)
*Stage-wise hyper-parameters $\left(N_{i},r_{i}\right)$ for CCM and $\left(N_{i},r_{i}^{\prime }\right)$ for DEF*		
Stage 1 (C=64)	$\left(N_{i}=1,r_{i}=8,r_{i}^{\prime }=8\right)$	
Stage 2 (C=128)	$\left(N_{i}=2,r_{i}=4,r_{i}^{\prime }=4\right)$	
Stage 3 (C=320)	$\left(N_{i}=4,r_{i}=4,r_{i}^{\prime }=2\right)$	
Stage 4 (C=512)	$\left(N_{i}=4,r_{i}=2,r_{i}^{\prime }=1\right)$	
*Decoder*		
$F_{\mathrm{fuse}}$	$\sum_{i}C_{i}\to 128$	1×1 Conv–BN–ReLU + 3×3 Conv–BN–ReLU
$F_{\mathrm{cls}}$	128→K	1×1 Conv for output logits
${\mathrm{Up}}_{\mathrm{ori}}$	—	Bilinear upsampling to input size H×W

*Note:* Italics indicate section headings that group related symbols and settings.

**Table 2:** Quantitative comparison on NYU Depth V2 under the NYU40 processed-label protocol. All metrics are reported as the mean ± sample standard deviation over three independent training runs. mIoU, F1, and OA denote mean Intersection over Union, mean F1-score, and Overall Accuracy, respectively. The best results are in bold.

Method	mIoU (%)	F1 (%)	Precision (%)	Recall (%)	OA (%)	Kappa (%)
ACNet [27]	44.318±0.382	58.985±0.374	61.961±1.665	58.827±0.689	72.897±0.652	69.722±0.633
SA-Gate [9]	47.278±0.251	61.956±0.039	64.472±0.958	61.605±0.833	74.530±0.673	71.648±0.706
PrimKD [15]	48.461±0.153	62.933±0.263	65.212±0.224	62.438±0.407	75.528±0.376	72.693±0.391
SGACNet [37]	45.218±0.174	59.645±0.151	63.922±1.021	58.434±1.084	73.361±0.261	70.258±0.246
ShapeConv [11]	47.209±0.190	61.480±0.220	64.600±0.366	60.261±0.232	74.824±0.768	71.944±0.800
AsymFormer [13]	48.734±0.210	63.304±0.319	65.242±0.057	62.767±0.668	75.127±0.492	72.303±0.506
SPGNet (Ours)	50.845±0.126	64.985±0.139	68.390±1.331	64.117±0.326	76.497±0.126	73.956±0.073

**Table 3:** Quantitative comparison on SUN RGB-D under the 37-class processed-label protocol. All metrics are reported as the mean ± sample standard deviation over three independent training runs. mIoU, F1, and OA denote mean Intersection over Union, mean F1-score, and Overall Accuracy, respectively. The best results are in bold.

Method	mIoU (%)	F1 (%)	Precision (%)	Recall (%)	OA (%)	Kappa (%)
ACNet [27]	43.946±0.230	58.039±0.152	61.135±0.957	56.229±0.428	79.098±0.112	75.381±0.130
PrimKD [15]	46.035±0.111	60.102±0.316	62.635±1.058	58.675±0.659	80.249±0.173	76.770±0.149
SA-Gate [9]	45.112±0.125	59.370±0.085	61.468±0.191	58.510±0.271	79.874±0.195	76.333±0.207
SGACNet [37]	44.269±0.158	58.256±0.382	61.897±1.256	57.361±0.931	79.312±0.139	75.703±0.123
ShapeConv [11]	45.385±0.149	59.506±0.553	63.318±0.554	56.905±0.734	79.916±0.340	76.308±0.411
AsymFormer [13]	47.558±0.268	61.533±0.313	64.139±0.721	60.165±0.089	80.640±0.133	77.273±0.159
SPGNet (Ours)	48.457±0.148	62.496±0.076	64.362±0.168	61.257±0.259	81.183±0.149	77.900±0.175

**Table 4:** Depth-only robustness comparison on NYU Depth V2. RGB images and labels remain unchanged, while only depth is corrupted by Gaussian noise or spatially shifted. Parentheses indicate the mIoU change from the clean result of the same fixed checkpoint; the best score under each condition is in bold.

Perturbation	Level	mIoU (%)						
		ACNet	SA-Gate	PrimKD	SGACNet	ShapeConv	AsymFormer	SPGNet
Clean	–	44.35	47.27	48.54	45.27	47.02	48.68	**50.78**
Gaussian noise	σ=0.05	44.17 (−0.18)	46.92 (−0.35)	48.21 (−0.33)	45.00 (−0.27)	46.84 (−0.18)	48.67 (−0.01)	**50.22** (−0.56)
	σ=0.10	43.89 (−0.46)	46.60 (−0.67)	47.95 (−0.59)	44.68 (−0.59)	46.08 (−0.94)	48.64 (−0.04)	**49.41** (−1.37)
	σ=0.20	43.41 (−0.94)	45.83 (−1.44)	47.33 (−1.21)	44.39 (−0.88)	43.10 (−3.92)	48.52 (−0.16)	**48.64** (−2.14)
Misalignment	1 pixel	44.38 (+0.03)	47.23 (−0.04)	48.53 (−0.01)	45.26 (−0.01)	46.98 (−0.04)	48.68 (+0.00)	**50.79** (+0.01)
	3 pixels	44.35 (+0.00)	47.18 (−0.09)	48.52 (−0.02)	45.25 (−0.02)	46.90 (−0.12)	48.66 (−0.02)	**50.78** (+0.00)
	5 pixels	44.28 (−0.07)	47.17 (−0.10)	48.43 (−0.11)	45.18 (−0.09)	46.79 (−0.23)	48.63 (−0.05)	**50.78** (+0.00)

**Table 5:** Ablation study on SUN RGB-D under the 37-class processed-label protocol.

Setting	CCM	DEF	mIoU (%)	F1 (%)	OA (%)	Kappa (%)
Baseline	–	–	45.395	59.309	79.749	76.239
Baseline+CCM	✓	–	46.375	60.271	80.183	76.755
Baseline+DEF	–	✓	45.817	59.651	79.483	76.009
SPGNet	✓	✓	**48.418**	**62.418**	**81.217**	**77.953**

*Note:* Bold values indicate the best results.

**Table 6:** Locally profiled complexity, runtime, and three-run mean Intersection over Union (mIoU) comparison of red–green–blue and depth (RGB-D) semantic segmentation methods appearing in the main quantitative experiments. Floating-point operations (FLOPs) are reported at 480×640; unsupported operators make the FLOP counts partial profiling results. End-to-end latency and frames per second (FPS) are measured for all methods on an NVIDIA GeForce RTX 5070 Ti with batch size 1 under the common input and timing protocol described in the text. All 122.21 M parameters reported for SPGNet are trainable.

Model	Backbone	Params (M)	FLOPs (G)	Latency (ms)	FPS	mIoU (%)	
						NYU Depth V2	SUN RGB-D
ACNet [27]	ResNet-50	116.63	116.11	20.45	48.89	44.318	43.946
SA-Gate [9]	ResNet-50	110.87	181.85	25.00	40.01	47.278	45.112
PrimKD [15]	MiT-B4	140.26	158.96	58.46	17.11	48.461	46.035
SGACNet [37]	R34-NBt1D	30.66	33.74	27.39	36.51	45.218	44.269
ShapeConv [11]	ResNeXt-101	109.98	169.16	28.25	35.40	47.209	45.385
AsymFormer [13]	official	33.06	36.22	13.39	74.66	48.734	47.558
SPGNet (Ours)	PVT-v2-B2 + ResNet34	122.21	99.91	34.64	28.87	**50.845**	**48.457**

*Note:* Bold values indicate the highest mIoU on each dataset.

**Table 7:** Controlled module-overhead profiling of the Cross-Modal Correction Module (CCM) and Dual-branch Enhancement Fusion Module (DEF). All configurations use identical encoders and decoder and are profiled on an NVIDIA GeForce RTX 5070 Ti with batch size 1 and 3×480×640 RGB and repeated-depth inputs.

Configuration	CCM	DEF	Params (M)	FLOPs (G)	Latency (ms)	Peak Memory (GB)
Baseline	–	–	50.82	53.81	14.44	0.45
Baseline+CCM	✓	–	88.13	67.93	23.57	0.69
Baseline+DEF	–	✓	84.91	85.80	24.75	0.66
SPGNet	✓	✓	122.21	99.91	34.64	0.91

## Data Availability

The NYU Depth V2 and SUN RGB-D datasets analyzed in this study were obtained from their official project websites: NYU Depth V2 (https://cs.nyu.edu/~fergus/datasets/nyu_depth_v2.html) and SUN RGB-D (https://rgbd.cs.princeton.edu/) (both accessed on 6 August 2026). The corresponding original dataset publications are cited in Refs. [2,3]. The source code and trained models of this study will be made available from the corresponding author upon reasonable request.

## Abbreviations

The following abbreviations are used in this manuscript:

RGB-D	Red–Green–Blue and Depth
SPGNet	Structure-Prior-Guided Network
CCM	Cross-Modal Correction Module
DEF	Dual-branch Enhancement Fusion Module
mIoU	Mean Intersection over Union
F1	F1-score
OA	Overall Accuracy
ACNet	Attention Complementary Network
SA-Gate	Separation-and-Aggregation Gate
PrimKD	Primary-Modality-Guided Knowledge Distillation
SGACNet	Spatial-Information-Guided Adaptive Context-Aware Network
ShapeConv	Shape-Aware Convolutional Layer
AsymFormer	Asymmetrical Cross-Modal Representation Learning

## References

[1] Long J., Shelhamer E., Darrell T. (2015). Fully Convolutional Networks for Semantic Segmentation. Proceedings of the IEEE Conference on Computer Vision and Pattern Recognition (CVPR).

[2] Silberman N., Hoiem D., Kohli P., Fergus R. (2012). Indoor Segmentation and Support Inference from RGBD Images. Proceedings of the European Conference on Computer Vision (ECCV).

[3] Song S., Lichtenberg S.P., Xiao J. (2015). SUN RGB-D: A RGB-D Scene Understanding Benchmark Suite. Proceedings of the IEEE Conference on Computer Vision and Pattern Recognition (CVPR).

[4] Gupta S., Girshick R., Arbeláez P., Malik J. (2014). Learning Rich Features from RGB-D Images for Object Detection and Segmentation. Proceedings of the European Conference on Computer Vision (ECCV).

[5] Hazirbas C., Ma L., Domokos C., Cremers D. (2016). FuseNet: Incorporating Depth into Semantic Segmentation via Fusion-Based CNN Architecture. Proceedings of the Asian Conference on Computer Vision (ACCV).

[6] Park S.J., Hong K.S., Lee S. (2017). RDFNet: RGB-D Multi-Level Residual Feature Fusion for Indoor Semantic Segmentation. Proceedings of the IEEE International Conference on Computer Vision (ICCV).

[7] Wang W., Neumann U. (2018). Depth-Aware CNN for RGB-D Segmentation. Proceedings of the European Conference on Computer Vision (ECCV).

[8] Jiao J., Wei Y., Jie Z., Shi H., Lau R.W.H., Huang T.S. (2019). Geometry-Aware Distillation for Indoor Semantic Segmentation. Proceedings of the IEEE/CVF Conference on Computer Vision and Pattern Recognition (CVPR).

[9] Chen X., Lin K.Y., Wang J., Wu W., Qian C., Li H., Zeng G. (2020). Bi-Directional Cross-Modality Feature Propagation with Separation-and-Aggregation Gate for RGB-D Semantic Segmentation. Proceedings of the European Conference on Computer Vision (ECCV).

[10] Zhang J., Liu H., Yang K., Hu X., Liu R., Stiefelhagen R. (2023). CMX: Cross-Modal Fusion for RGB-X Semantic Segmentation With Transformers. IEEE Trans. Intell. Transp. Syst..

[11] Cao J., Leng H., Lischinski D., Cohen-Or D., Tu C., Li Y. (2021). ShapeConv: Shape-Aware Convolutional Layer for Indoor RGB-D Semantic Segmentation. Proceedings of the IEEE/CVF International Conference on Computer Vision (ICCV).

[12] Wang Y., Chen X., Cao L., Huang W., Sun F., Wang Y. (2022). Multimodal Token Fusion for Vision Transformers. Proceedings of the IEEE/CVF Conference on Computer Vision and Pattern Recognition (CVPR).

[13] Du S., Wang W., Guo R., Wang R., Tang S. (2024). AsymFormer: Asymmetrical Cross-Modal Representation Learning for Mobile Platform Real-Time RGB-D Semantic Segmentation. Proceedings of the IEEE/CVF Conference on Computer Vision and Pattern Recognition Workshops (CVPRW).

[14] Yin B., Zhang X., Li Z., Liu L., Cheng M.M., Hou Q. DFormer: Rethinking RGBD Representation Learning for Semantic Segmentation. Proceedings of the International Conference on Learning Representations (ICLR).

[15] Hao Z., Xiao Z., Luo Y., Guo J., Wang J., Shen L., Hu H. (2024). PrimKD: Primary Modality Guided Multimodal Fusion for RGB-D Semantic Segmentation. Proceedings of the ACM International Conference on Multimedia (ACM MM).

[16] Yin B.W., Cao J.L., Cheng M.M., Hou Q. (2025). DFormerv2: Geometry Self-Attention for RGBD Semantic Segmentation. Proceedings of the IEEE/CVF Conference on Computer Vision and Pattern Recognition (CVPR).

[17] Gupta S., Arbeláez P., Malik J. (2013). Perceptual Organization and Recognition of Indoor Scenes from RGB-D Images. Proceedings of the IEEE Conference on Computer Vision and Pattern Recognition (CVPR).

[18] Eigen D., Fergus R. (2015). Predicting Depth, Surface Normals and Semantic Labels with a Common Multi-Scale Convolutional Architecture. Proceedings of the IEEE International Conference on Computer Vision (ICCV).

[19] Deng Z., Todorovic S., Latecki L.J. (2015). Semantic Segmentation of RGBD Images with Mutex Constraints. Proceedings of the IEEE International Conference on Computer Vision (ICCV).

[20] Li Z., Gan Y., Liang X., Yu Y., Cheng H., Lin L. (2016). LSTM-CF: Unifying Context Modeling and Fusion with LSTMs for RGB-D Scene Labeling. Proceedings of the European Conference on Computer Vision (ECCV).

[21] Wang J., Wang Z., Tao D., See S., Wang G. (2016). Learning Common and Specific Features for RGB-D Semantic Segmentation with Deconvolutional Networks. Proceedings of the European Conference on Computer Vision (ECCV).

[22] He Y., Chiu W.C., Keuper M., Fritz M. (2017). STD2P: RGBD Semantic Segmentation Using Spatio-Temporal Data-Driven Pooling. Proceedings of the IEEE Conference on Computer Vision and Pattern Recognition (CVPR).

[23] Qi X., Liao R., Jia J., Fidler S., Urtasun R. (2017). 3D Graph Neural Networks for RGBD Semantic Segmentation. Proceedings of the IEEE International Conference on Computer Vision (ICCV).

[24] Cheng Y., Cai R., Li Z., Zhao X., Huang K. (2017). Locality-Sensitive Deconvolution Networks with Gated Fusion for RGB-D Indoor Semantic Segmentation. Proceedings of the IEEE Conference on Computer Vision and Pattern Recognition (CVPR).

[25] Jiang J., Zheng L., Luo F., Zhang Z. (2018). RedNet: Residual Encoder-Decoder Network for Indoor RGB-D Semantic Segmentation. arXiv.

[26] Xu D., Ouyang W., Wang X., Sebe N. (2018). PAD-Net: Multi-Tasks Guided Prediction-and-Distillation Network for Simultaneous Depth Estimation and Scene Parsing. Proceedings of the IEEE/CVF Conference on Computer Vision and Pattern Recognition (CVPR).

[27] Hu X., Yang K., Fei L., Wang K. (2019). ACNet: Attention Based Network to Exploit Complementary Features for RGBD Semantic Segmentation. Proceedings of the IEEE International Conference on Image Processing (ICIP).

[28] Zhou H., Qi L., Wan Z., Huang H., Yang X. (2020). RGB-D Co-Attention Network for Semantic Segmentation. Proceedings of the Asian Conference on Computer Vision (ACCV).

[29] Xing Y., Wang J., Zeng G. (2020). Malleable 2.5D Convolution: Learning Receptive Fields along the Depth-axis for RGB-D Scene Parsing. Proceedings of the European Conference on Computer Vision (ECCV).

[30] Lin D., Zhang R., Ji Y., Li P., Huang H. (2020). SCN: Switchable Context Network for Semantic Segmentation of RGB-D Images. IEEE Trans. Cybern..

[31] Lin D., Huang H. (2020). Zig-Zag Network for Semantic Segmentation of RGB-D Images. IEEE Trans. Pattern Anal. Mach. Intell..

[32] Wang Y., Huang W., Sun F., Xu T., Rong Y., Huang J. (2020). Deep Multimodal Fusion by Channel Exchanging. Proceedings of the Advances in Neural Information Processing Systems (NeurIPS).

[33] Chen L.Z., Lin Z., Wang Z., Yang Y.L., Cheng M.M. (2021). Spatial Information Guided Convolution for Real-Time RGB-D Semantic Segmentation. IEEE Trans. Image Process..

[34] Seichter D., Köhler M., Lewandowski B., Wengefeld T., Gross H.M. (2021). Efficient RGB-D Semantic Segmentation for Indoor Scene Analysis. Proceedings of the IEEE International Conference on Robotics and Automation (ICRA).

[35] Yang E., Zhou W., Qian X., Yu L. (2022). MGCNet: Multilevel Gated Collaborative Network for RGB-D Semantic Segmentation of Indoor Scene. IEEE Signal Process. Lett..

[36] Zhou W., Yang E., Lei J., Wan J., Yu L. (2023). PGDENet: Progressive Guided Fusion and Depth Enhancement Network for RGB-D Indoor Scene Parsing. IEEE Trans. Multimed..

[37] Zhang Y., Xiong C., Liu J., Ye X., Sun G. (2023). Spatial-Information Guided Adaptive Context-Aware Network for Efficient RGB-D Semantic Segmentation. IEEE Sens. J..

[38] Yang J., Bai L., Sun Y., Tian C., Mao M., Wang G. (2024). Pixel Difference Convolutional Network for RGB-D Semantic Segmentation. IEEE Trans. Circuits Syst. Video Technol..

[39] Bai L., Yang J., Tian C., Sun Y., Mao M., Xu Y., Xu W. (2025). DCANet: Differential Convolution Attention Network for RGB-D Semantic Segmentation. Pattern Recognit..

[40] Wu Z., Zhou Z., Allibert G., Stolz C., Demonceaux C., Ma C. (2024). Transformer Fusion for Indoor RGB-D Semantic Segmentation. Comput. Vis. Image Underst..

[41] Ying X., Chuah M.C. (2022). UCTNet: Uncertainty-Aware Cross-Modal Transformer Network for Indoor RGB-D Semantic Segmentation. Proceedings of the European Conference on Computer Vision (ECCV).

[42] Zhang J., Liu R., Shi H., Yang K., Reiß S., Peng K., Fu H., Wang K., Stiefelhagen R. (2023). Delivering Arbitrary-Modal Semantic Segmentation. Proceedings of the IEEE/CVF Conference on Computer Vision and Pattern Recognition (CVPR).

[43] Dong S., Feng Y., Yang Q., Huang Y., Liu D., Fan H. (2024). Efficient Multimodal Semantic Segmentation via Dual-Prompt Learning. Proceedings of the IEEE/RSJ International Conference on Intelligent Robots and Systems (IROS).

[44] Ge M., Su W., Gao J., Jia G. (2025). CDMANet: Central Difference Mutual Attention Network for RGB-D Semantic Segmentation. J. Supercomput..

[45] Cai J., Su J., Li Q., Yang W., Wang S., Zhao T., He S., Liu W. (2025). Keep the Balance: A Parameter-Efficient Symmetrical Framework for RGB+X Semantic Segmentation. Proceedings of the IEEE/CVF Conference on Computer Vision and Pattern Recognition (CVPR).

